# EMUlator: An Elementary Metabolite Unit (EMU) Based Isotope Simulator Enabled by Adjacency Matrix

**DOI:** 10.3389/fmicb.2019.00922

**Published:** 2019-04-30

**Authors:** Chao Wu, Chia-hsin Chen, Jonathan Lo, William Michener, PinChing Maness, Wei Xiong

**Affiliations:** ^1^National Renewable Energy Laboratory, Golden, CO, United States; ^2^Institute of Nuclear Energy Research, Taoyuan, Taiwan

**Keywords:** adjacency matrix, elementary metabolite unit (EMU), fractional labeling (FL), *Clostridium acetobutylicum*, phosphoketolase

## Abstract

Stable isotope based metabolic flux analysis is currently the unique methodology that allows the experimental study of the integrated responses of metabolic networks. This method primarily relies on isotope labeling and modeling, which could be a challenge in both experimental and computational biology. In particular, the algorithm implementation for isotope simulation is a critical step, limiting extensive usage of this powerful approach. Here, we introduce *EMUlator* a Python-based isotope simulator which is developed on Elementary Metabolite Unit (EMU) algorithm, an efficient and powerful algorithm for isotope modeling. We propose a novel adjacency matrix method to implement EMU modeling and exemplify it stepwise. This method is intuitively straightforward and can be conveniently mastered for various customized purposes. We apply this arithmetic pipeline to understand the phosphoketolase flux in the metabolic network of an industrial microbe *Clostridium acetobutylicum*. The resulting design enables a high-throughput and non-invasive approach for estimating phosphoketolase flux *in vivo*. Our computational insights allow the systematic design and prediction of isotope-based metabolic models and yield a comprehensive understanding of their limitations and potentials.

## Introduction

^13^C metabolic flux analysis (MFA) is currently the only experimental methodology to quantitatively understand intracellular biochemical networks by means of stable isotope tracing, labeling pattern analysis and indispensably, metabolic modeling which is based on mass and isotope balancing (Stephanopoulos, 1999; Sauer, 2006). Importantly, isotope modeling can provide additional key information that further defines the system, enabling quantification of the fluxes in parallel or cyclic pathways that cannot be estimated reliably by mass balance only. A variety of mathematical models were developed to establish relationship between isotope distribution and metabolic flux, including isotopomers (Schmidt et al., 1997), cumomers (Wiechert et al., 1999), and bondomers (Van Winden et al., 2002). However, previous methods suffer from the computational challenge in resolving realistic and large-scale metabolic network, as a large number of isotopomer equations need to be solved.

To address this limitation, a creative computational framework based on Elementary Metabolite Unit (EMU) was proposed (Antoniewicz et al., 2007a). The EMUs of a metabolite are defined as the non-empty subsets of all that compound's atoms (usually carbon atom). EMU can be cataloged by size, i.e., the number of atoms in it. With atom transition map, this framework will trace and identify the minimal relevant metabolic information needed to simulate isotope patterns and solve the optimization problem, and therefore greatly reduces the number of balance equations and computation burden, i.e., 95% reduction of the variables needed for the simulation in an *E. coli* model without any loss of information (Antoniewicz et al., 2007b). To date, EMU algorithm has garnered increasing attention in metabolic analysis (Quek et al., 2009; Sokol et al., 2012; Weitzel et al., 2013; Kajihata et al., 2014; Shupletsov et al., 2014; Young, 2014). Better leveraging this approach in understanding cell metabolism will require the development of novel computational packages which are easy to program and can fit new and broad application scenarios. However, to date, computational approaches that can straightforwardly and efficiently implement the EMU algorithm are still insufficient.

Here, we develop a new computational toolbox for steady state metabolic modeling analysis, the *EMUlator*, which accomplishes EMU modeling through an adjacency matrix-based approach. In graph theory, an adjacency matrix is used to quantitatively represent a graph, of which the elements indicate the connectivity of vertex pairs in rows and columns. Essentially, metabolic network is a directed graph with branches, thus it can be transformed into adjacency matrix for the ease of programming. Utilizing adjacency matrix, the *EMUlator* can efficiently simulate isotope distributions for ^13^C-MFA. To demonstrate its functionality, we decompose the EMUs for isotope simulation in a tricarboxylic acid (TCA) cycle which represents a realistic metabolic network. Furthermore, we applied this newly developed software in modeling and analyzing the flux of phosphoketolase pathway in *Clostridium acetobutylicum* xylose catabolism. By decomposing the network and simulating metabolite isotopomer patterns, we found a good correlation between phosphoketolase flux and the fractional labeling of acetate, which has never been characterized in an isotope tracer experiment. Coupled with GC-MS analysis of acetate, this EMUlator-enabled analysis leads to a novel and high-throughput methodology for quantitatively understanding the phosphoketolase pathway in response to environmental and genetic perturbation. As exemplified, *EMUlator* aims to be a universal and powerful tool for isotope tracer modeling and for gaining quantitative understanding of cell metabolism. The software and its instruction are available at Supplementary Information.

## Results

### Overview of the *EMUlator* Pipeline

The *EMUlator* pipeline is designed in Python, capable of performing a complete isotope simulation and prediction of a metabolic network for ^13^C-MFA. Previous tools, such as Metran (Antoniewicz et al., 2006, 2007a), OpenFlux (Quek et al., 2009; Shupletsov et al., 2014) and INCA (Young, 2014), are able to perform such modeling, however based on Matlab platform which is not an open and free computing environment. In addition, previous EMU modeling was substantially less transparent than the one we present here. A key distinguishing feature of *EMUlator* is the usage of adjacency matrix. This ensures a graphic expression of the algorithm which can be understood intuitively and implemented iteratively. In particular, *EMUlator* provides a more detailed and principled procedure of EMU modeling, which decomposes metabolic network into EMU reactions, sets up EMU balances and simulates labeling distribution. Most importantly, the EMU deconstruction results in the reduction of the metabolic network model leading to a smaller set of EMU reactions which preserves all the information contained in it but decreases running time significantly.

### *EMUlator* Transforms Metabolic Network Into Adjacency Matrix

**Figure 1:**
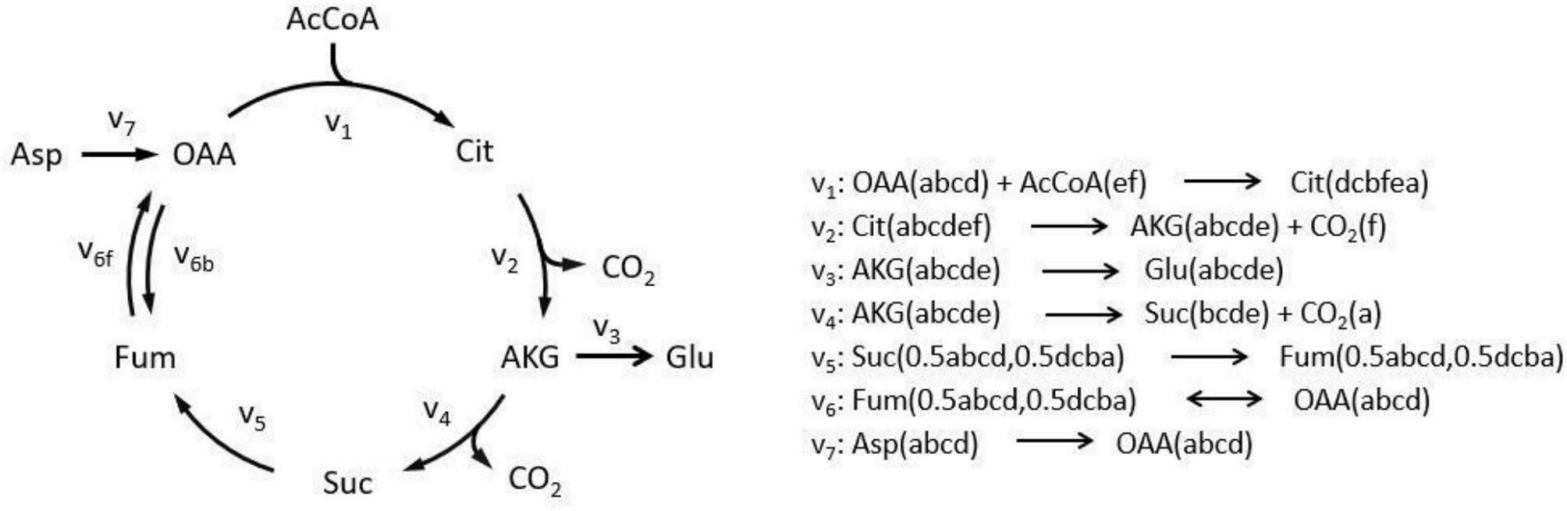
Simplified tricarboxylic acid (TCA) cycle to illustrate adjacency matrix-based EMU decomposition. Reactions involved in the metabolic model are listed on the right. Lowercase letters in brackets demonstrate atom transition in each reaction. Decimals indicate EMU equivalents due to rotation axis within molecule. AcCoA, acetyl coenzyme A; AKG, α-ketoglutarate; Asp, aspartate; Cit, citrate; Fum, fumarate; Glu, glutamate; OAA, oxaloacetate; Suc, succinate; subscript f, forward reaction; subscript b, backward reaction.

**Figure 2:**
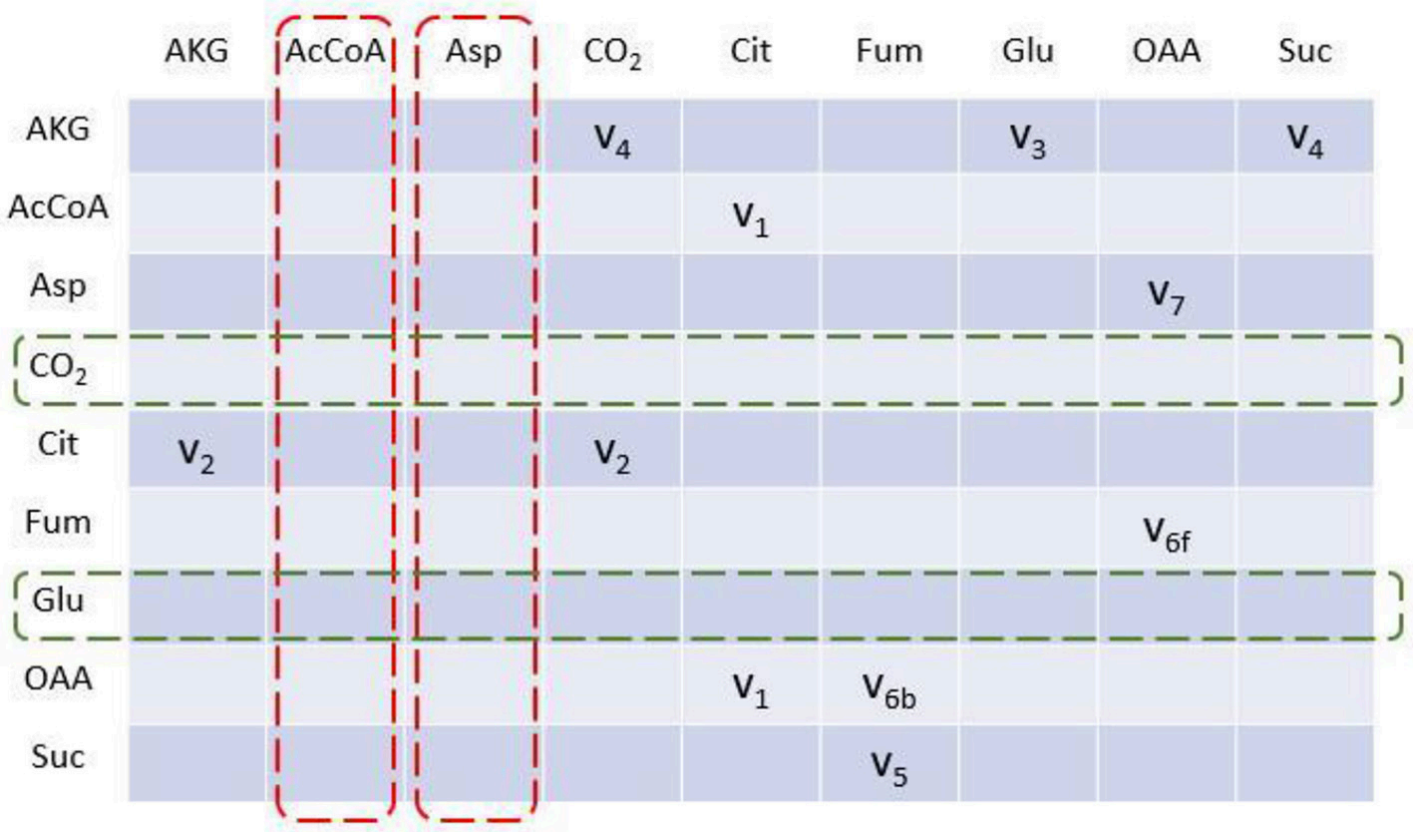
Metabolite adjacency matrix (MAM) of the TCA cycle network. MAM is a square matrix with all involved metabolites on both rows and columns. Each element indicates reaction(s) through which reactant (row metabolite) is converted into product (column metabolite). Metabolites with no element in column are identified as initial substrates (dashed red box); metabolites with no element in row are identified as final products (dashed green box).

To illustrate algorithm of the program comparably, we implement TCA cycle model as an example, as this representative metabolic network was also used in the original EMU work (Antoniewicz et al., 2007a). In this network, aspartate and acetyl coenzyme A (AcCoA) are the substrates, while CO_2_ and glutamate are final products. Reactions with carbon atom transitions are listed in Figure 1. As a directed graph, any metabolic network with branches (due to cleavage and condensation reactions) can be transformed into a metabolite adjacency matrix (MAM). All metabolites are grouped in both row and column coordinates, thus forming a square matrix. Row metabolites appear as reactants while column metabolites are products of each reactions. Elements determined by row and column coordinates are connecting reactions for reactants and products. Reaction in element may not be unique because identical reactant and product could be involved in different reactions. As such, inputs and outputs of the network are easy to identify. Herein, columns without element are identified as substrates since they have no precursors (i.e., columns for AcCoA and Aspartate in dashed red boxes, Figure 2), while rows without element are identified as final products (i.e., rows for CO_2_ and glutamate in dashed green boxes, Figure 2). Overall, MAM reflects the connectivity of metabolites in a network.

### *EMUlator* Decomposes MAM Into EMU Adjacency Matrix (EAM)

**Figure 3:**
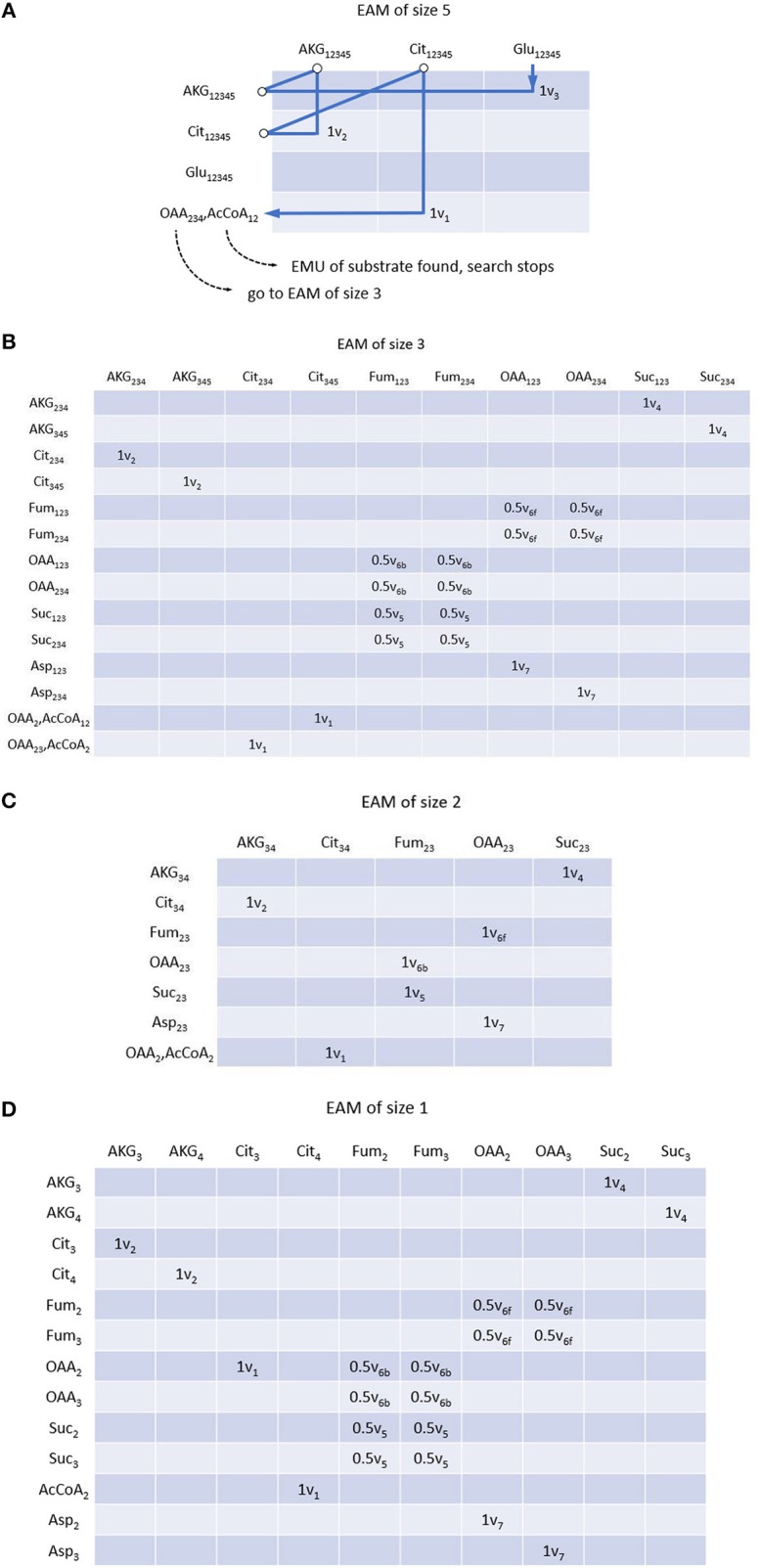
EMU adjacency matrix (EAM) of size 5 **(A)**, size 3 **(B)**, size 2 **(C)** and size 1 **(D)**. Each element indicates reaction(s) through which reactant (row metabolite) is converted into product (column metabolite) as well as reaction coefficients. Construction of EAM starts from the EMU to be simulated, i.e., Glu_12345_ in this example. All precursors of corresponding EMU can be found with MAM and atom transition using breadth-first search. Precursor searching continues until initial substrates. If condensation reactions are encountered, precursors of smaller size are considered as new start points, respectively. EMUs of same size are located in the same EAM.

EMU decomposition of a metabolic network can start at the size of EMU(s) that need to be simulated. In this example, we simulate the Mass Distribution Vector (MDV, the fractional abundance of each isotopolog normalized to the sum of all possible isotopologues.) (Nanchen et al., 2007) of glutamate Glu_12345_ (size 5). All EMU reactions that are needed for this simulation can be identified iteratively via MAM. Glu_12345_ as a product can be found in the column of EAM (size 5) (see Figure 3A), its precursor AKG_12345_ is illustrated in the corresponding row through the reaction V_3_. Similarly, for the product AKG_12345_, we can locate its precursor Cit_12345_ via v_2_. Lastly for the product (in column) Cit_12345_, we identify reaction v_1_, in which both OAA_234_ and AcCoA_12_ are the reactants. Since EMUs of smaller size are identified in condensation reactions, they will be used as new start points for searching. Therefore, we can follow the OAA_234_ (AcCoA_12_ is identified as an EMU of the substrate, and thus the searching stops), and search all other EMUs at size 3 (Figure 3B). All precursor EMUs for multiple precursors [e.g., due to equivalent EMUs (Antoniewicz et al., 2007a)], can be identified through breadth-first search. As such, adjacent matrix provides a straightforward and iterative path allowing us to trace back EMUs of smaller sizes until the EMUs of network substrates are identified (i.e., Aspartate and AcCoA in this example). Once all set of the EMUs are obtained, EMUs can be arranged into different EAMs per size. Similar to MAM, row and column coordinates of EAM correspond to reactants and products of each reaction, respectively, with the difference that EMUs subject to convolution also appear in rows of EAM, and the coefficients of an element equal to the stoichiometric coefficients of corresponding reactant and product. Complete EAMs after EMU decomposition of the example are shown in Figure 3.

### *EMUlator* Significantly Reduces the Size of EAMs

**Figure 4:**
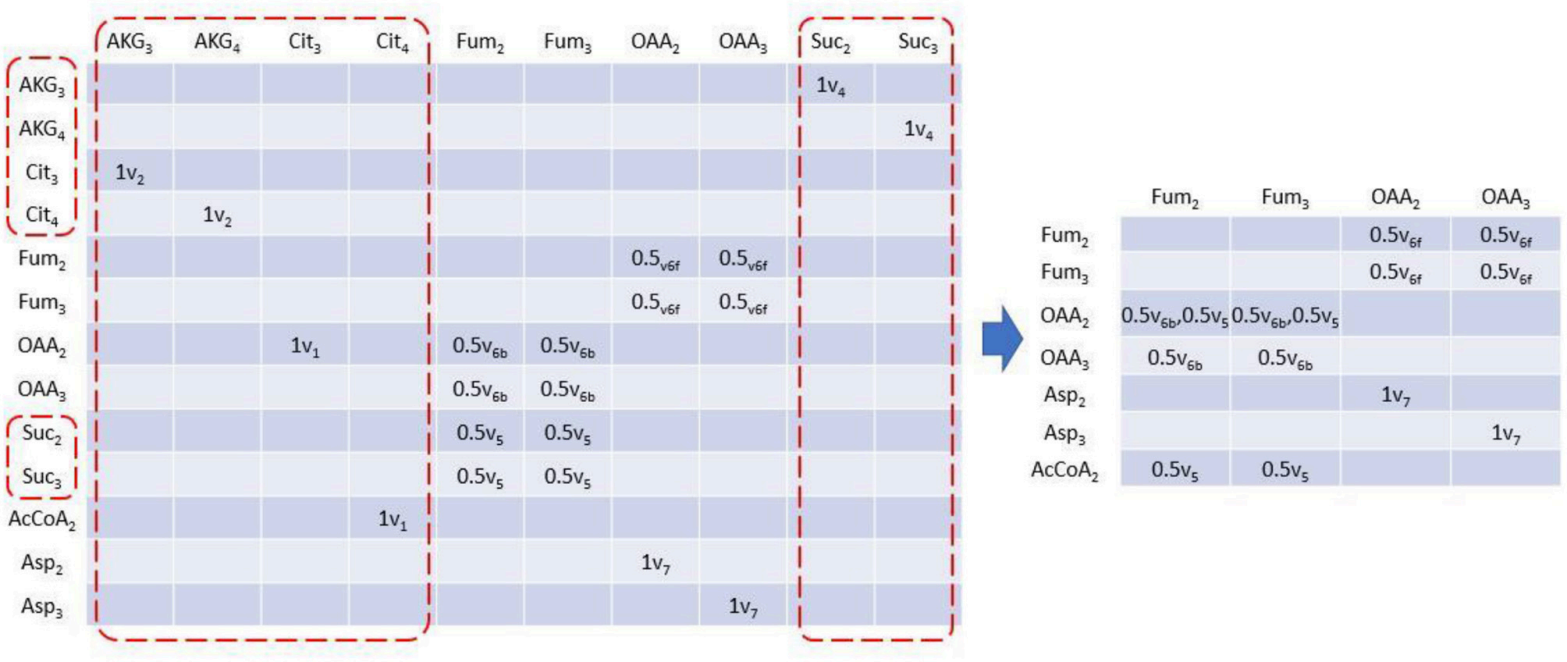
Reduced EAM of size 1. Column metabolites with only one element can be lumped because it has solo influx. These columns can be eliminated therefore, and their corresponding row metabolites will be replaced by its precursor. Elimination and replacement occur iteratively until no solo element column exists. Rows with identical metabolite will be combined.

*EMUlator* can also reduce the scale of EAM. In steady state isotope modeling, labeling pattern can only be modified by convolution of two or more metabolites and unimolecular reactions may be lumped without affecting simulations which helps to reduce the scale of EAM. Unimolecular reactions can be easily identified in EAM as those with solo element in a column, which means its corresponding product only has a single source. Those columns will be deleted with identical metabolites in rows all renamed by their precursors. For example, in EAM of size 1, after AKG_3_ column is eliminated, AKG_3_ in row will be renamed with Cit_3_ which produces AKG_3_ by reaction v_2_ (Figure 4). Moreover, since Cit_3_ is still from a unimolecular reaction (v_1_), we eliminate the column as well and Cit_3_ in row will be renamed by its precursor OAA_2_ via v_1_. The deletion of the column and renaming of corresponding row continue until there is no solo-element column in EAM. Finally, multiple rows with identical metabolite name will be combined, indicating the identical reactant and product are connected by different reactions.

### *EMUlator* Identifies and Combines Equivalent EMUs

**Figure 5:**
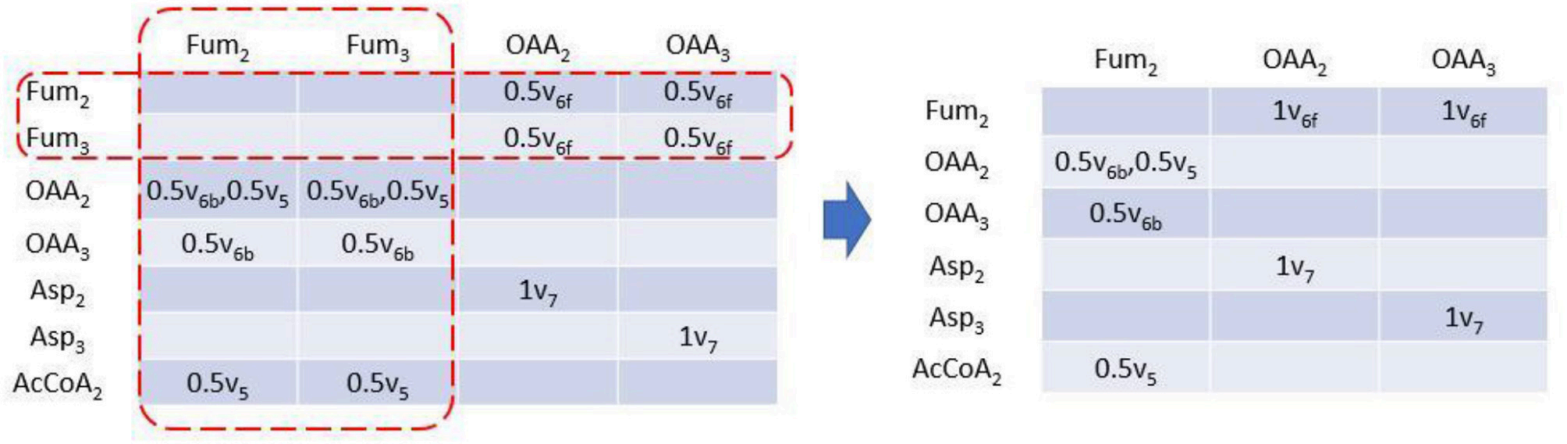
EAM of size 1 with EMU equivalents combined. EMU equivalents can be combined. Coefficients of equivalent columns are combined and divided by the number of EMU equivalents. Coefficients of equivalent rows are just combined.

Rotationally symmetric molecules (i.e., fumaric acid and succinic acid) will give rise to equivalent EMUs (Antoniewicz et al., 2007a), which are undistinguishable for enzyme, and react identically in the reactions. EMUlator can combine those EMUs as they will have the same probability to get certain labeling pattern. Metabolites which could generate equivalent EMUs are indicated with fractional carbon atoms in Figure 1. Fum_2_ and Fum_3_ are equivalent EMUs of size 1 in this example (Figure 5). Element coefficients of row equivalent EMUs are combined, while coefficients of column equivalent EMUs are combined and divided by the number of equivalents. Eventually, EMU variables were reduced from 24 of initial EMU model to 9 after lumping unimolecular reactions and combining equivalent EMU, yielding the same results as the original EMU work (Antoniewicz et al., 2007a).

### *EMUlator* Establishes EMU Balances From EAMs

To simulate the labeling pattern of a given metabolite, EMU balances are established from EMU reaction subnetworks of different size, and MDVs can be calculated according to:

$$\begin{array}{l} X_{i}=A_{i}^{-1}\cdot B_{i}\cdot Y_{i} \end{array}$$

**Figure 6:**
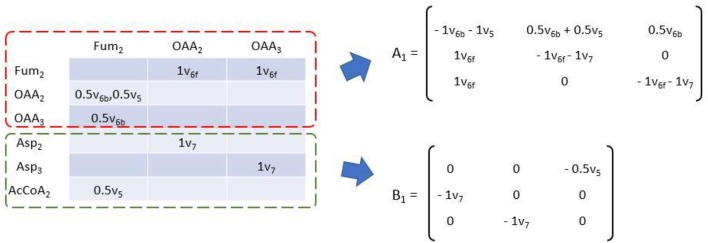
Transformation of EAM to establish EMU balance of size 1. Matrix function A of flux variables can be obtained by transposition of the upper square submatrix (dashed red box) with negative sums of each column of original EAM as diagonal elements. Matrix function B can be obtained by transposition of the lower submatrix (dashed green box) with all elements taken opposite sign.

Where A_i_ and B_i_ are matrix function of flux variable of size i. A_i_ is square matrix whose shape is dependent on the number of EMU balance m of current size. B_i_'s shape is m × n where n is the number of available EMU variables (Antoniewicz et al., 2007a). Simulation starts from size 1, and Y_1_ are MDVs of network substrates. Other MDVs are calculated and then used in Y of larger size. A_i_ and B_i_ can be easily deduced from EAM as demonstrated in Figure 6. Diagonal elements of EAM are first set to be the negative sum of other elements of current column. Then the transposed upper square submatrix will be A_1_, and the lower submatrix will be B_1_ after transposition and all elements negated. The transformation is made according to the isotopomer balance which states that the sum of all influx to an EMU multiplied by its MDV (∑*v*_*i*_ · *MDV*) is equal to the sum of the individual product of each influx v_i_ multiplied by MDV_i_ (∑(*v*_*i*_. *MDV*_*i*_)). Apparently, diagonal element represents the total influx of corresponding EMU. Multiplied by MDV of balanced EMU, the product is equal to the sum of all labeling pattern sources, including those unknown (X_i_) and known (Y_i_) EMU variables. EMU balances of larger size can be established likewise until the Glu_12345_ are eventually simulated. EMU balances of all sizes are shown below:

EMU balance of size 1

$$\begin{array}{l} \left[\begin{array}{ccc} -v_{6b}-v_{5} & 0.5v_{6b}+\;0.5v_{5} & 0.5v_{6b}\\ v_{6f} & -v_{6f}-v_{7} & 0\\ v_{6f} & 0 & -v_{6f}-v_{7} \end{array}\right]\cdot \left[\begin{array}{c} MDV_{Fum_{2}}\\ MDV_{OAA_{2}}\\ MDV_{OAA_{3}} \end{array}\right]\\ \;=\left[\begin{array}{ccc} 0 & 0 & -0.5v_{5}\\ -v_{7} & 0 & 0\\ 0 & -v_{7} & 0 \end{array}\right]\cdot \left[\begin{array}{c} MDV_{Asp_{2}}\\ MDV_{Asp_{3}}\\ MDV_{AcCoA_{2}} \end{array}\right] \end{array}$$

EMU balance of size 2

$$\begin{array}{l} \left[\begin{array}{cc} -v_{6b}-v_{5} & v_{6b}\\ v_{6f} & -v_{6f}-v_{7} \end{array}\right]\cdot \left[\begin{array}{c} MDV_{Fum_{23}}\\ MDV_{OAA_{23}} \end{array}\right]\text{=}\left[\begin{array}{cc} 0 & v_{5}\\ -v_{7} & 0 \end{array}\right]\\ \;\cdot \left[\begin{array}{c} MDV_{Asp_{23}}\\ MDV_{OAA_{2}}\times MDV_{AcCoA_{2}} \end{array}\right] \end{array}$$

EMU balance of size 3

$$\begin{array}{l} \;[\begin{array}{ccc} -v_{6b}-v_{5} & 0.5v_{6b} & 0.5v_{6b}\\ v_{6f} & -v_{6f}-v_{7} & 0\\ v_{6f} & 0 & -v_{6f}-v_{7} \end{array}]\cdot \left[\begin{array}{c} MDV_{Fum_{123}}\\ MDV_{OAA_{123}}\\ MDV_{OAA_{234}} \end{array}\right]\text{=}\\ \left[\begin{array}{cccc} \begin{array}{c} -0.5v_{5}\\ 0\\ 0 \end{array} & \begin{array}{c} -0.5v_{5}\\ 0\\ 0 \end{array} & \begin{array}{c} 0\\ -v_{7}\\ 0 \end{array} & \begin{array}{c} 0\\ 0\\ -v_{7} \end{array} \end{array}\right]\cdot \left[\begin{array}{c} MDV_{OAA_{2}}\times MDV_{AcCoA_{12}}\\ MDV_{OAA_{23}}\times MDV_{AcCoA_{2}}\\ MDV_{Asp_{123}}\\ MDV_{Asp_{234}} \end{array}\right] \end{array}$$

EMU balance of size 4

$$\begin{array}{l} \left[-v_{3}\right]\cdot \left[MDV_{Glu_{12345}}\right]=\left[-v_{3}\right]\cdot \left[MDV_{OAA_{123}}\times MDV_{AcCoA_{12}}\right] \end{array}$$

### *EMUlator* Designs Optimal ^13^C-Tracer Experiment for Quantifying Metabolic Flux of Interest

To demonstrate the performance of *EMUlator* in a large-scale setting that reflects the complexity of realistic cell metabolism, we next performed simulation of isotope distributions in a larger network model of *C. acetobutylicum*. *C. acetobutylicum* is a solventogenic clostridium and represents a promising chassis microbe capable of utilizing lignocellulose-derived pentose sugar (i.e., xylose) for biofuels production (Mitchell, 1998; Gu et al., 2011). In this case, pentose catabolism through phosphoketolase pathway (Grimmler et al., 2010; Servinsky et al., 2010; Liu et al., 2012) is of special interest in that this pathway has been recognized as a key target for constructing synthetic pathways (e.g., Non-oxidative glycolysis) that bypass CO_2_ loss via pyruvate decarboxylase and thus enhance carbon yield in final products (Bogorad et al., 2013). Here we used the adjacency matrix-based *EMUlator* to simulate labeling patterns of metabolites and show how it facilitates the selection of best isotope substrates and readouts quantifying the *in vivo* phosphoketolase activity.

**Figure 7:**
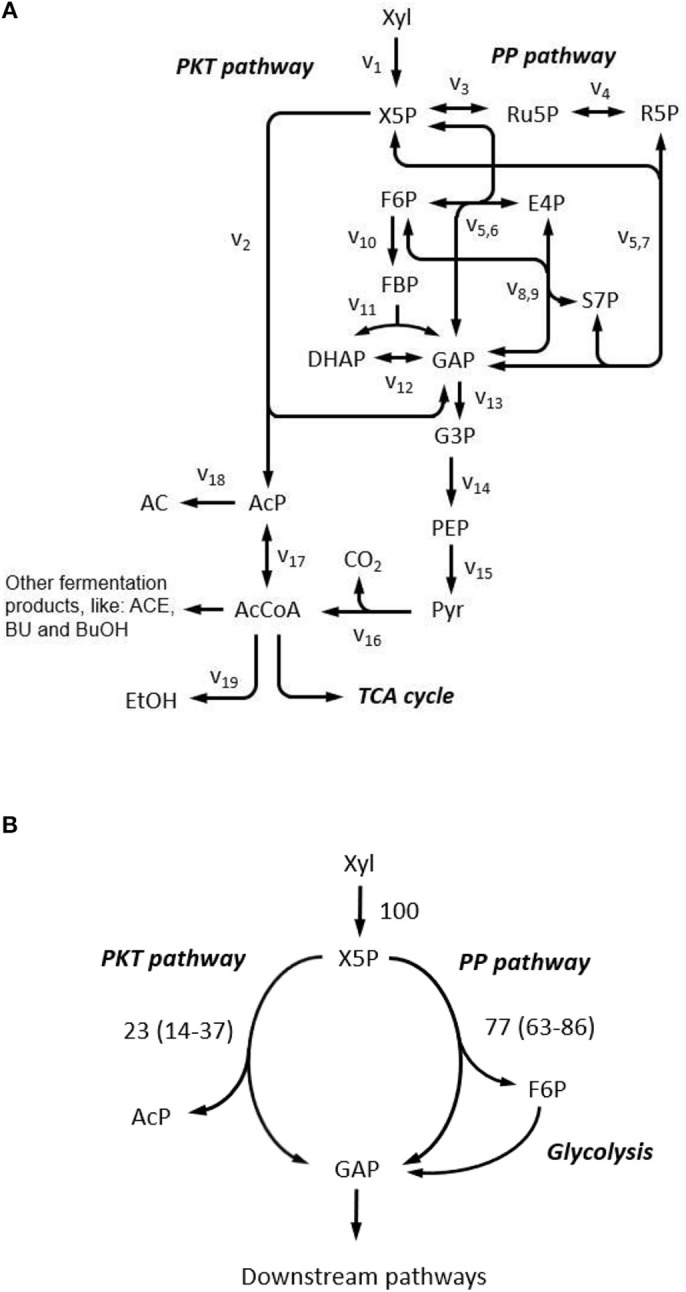
Xylose metabolism in *C. acetobutylicum*. **(A)** Reaction network constructed for xylose metabolism. Complete reaction list is provided in Supplementary Table 1. AC, acetate; ACE, acetone; AcP, Acetyl phosphate; BU, butyrate; BuOH, butanol; DHAP, dihydroxyacetone phosphate; E4P, erythrose-4-phosphate; EtOH, ethanol; F6P, fructose-6-phosphate; FBP, fructose-1,6-bisphosphate; G3P, 3-phosphoglycerate; GAP, glyceraldehyde-3-phosphate; PEP, phosphoenolpyruvate; Pyr, pyruvate; R5P, ribose-5-phosphate; Ru5P, ribulose-5-phosphate; S7P, sedoheptulose-7-phosphate; X5P, xylulose-5-phosphate; Xyl, xylose; PKT pathway, phosphoketolase pathway; PP pathway, pentose phosphate pathway; TCA cycle, tricarboxylic acid cycle. **(B)** Metabolic flux ratio through phosphoketolase at 5 g L^−1^ xylose. Flux ratios are relative to total xylose uptake rate, and values are presented as the mean of two replicates and 95% confidence intervals are provided in the parentheses.

First, a biochemical network for xylose metabolism of *C. acetobutylicum* was constructed, based on the genome information (Nölling et al., 2001; Bao et al., 2011). As shown in Figure 7A, after phosphorylation, xylose can be metabolized either through the non-oxidative pentose phosphate pathway or cleaved by phosphoketolase to form acetyl-phosphate and glyceraldehyde-3-phosphate. Acetyl-phosphate is further directed to generate extracellular fermentative products (i.e., acetate, ethanol, acetone, butanol, butyrate) and glyceraldehyde-3-phosphate can enter pentose phosphate pathway in which reactions are highly reversible due to the nature of isomerase, epimerase, transketolase, and transaldolase. The oxidative pentose phosphate pathway is not considered which was verified to be inactive in *C. acetobutylicum* (Au et al., 2014). The TCA cycle is not included as it does not influence the labeling patterns of the upstream metabolites.

**Figure 8:**
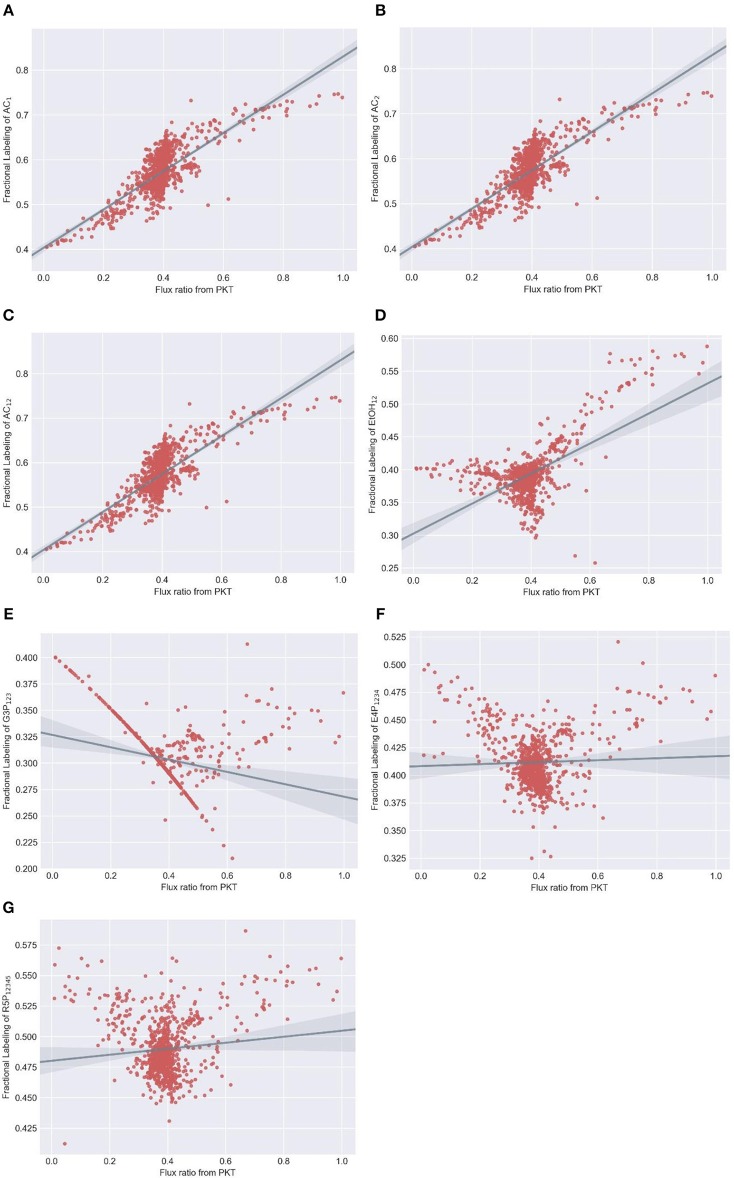
Simulated fractional labeling (FL) of metabolites at different flux ratio from phosphoketolase with 100% 1,2-^13^C xylose as substrate. Random fluxes are generated 1000 times subjecting to xylose metabolism network. MDVs of **(A)** AC_1_, **(B)** AC_2_, **(C)** AC_12_, **(D)** EtOH_12_, **(E)** G3P_123_, **(F)** E4P_1234_ and **(G)** R5P_12345_ are simulated using adjacency matrix-based EMU decomposition method proposed in this work. Metabolite FLs and flux ratio from PKT are subsequently calculated and plotted correspondingly. Regression line and 95% confidence intervals are also plotted.

We selected acetate (AC), ethanol, 3-phosphoglycerate, erythrose-4-phosphate and ribose-5-phosphate as candidate readouts for reflecting phosphoketolase activity since MDVs of these metabolites can be experimentally obtained either from direct determination or derivation from amino acid MDVs (Nanchen et al., 2007). Meanwhile, we tested all commercially available xylose tracers: 1-^13^C xylose, 2-^13^C xylose, 3-^13^C xylose, 4-^13^C xylose, 5-^13^C xylose, 1,2-^13^C xylose, U-^13^C xylose. MDVs were simulated using *EMUlator* in all possible combinations of these candidate readouts and tracers. Goodness of correlation between Fractional Labeling (FL) (defined in Materials and Methods) and flux ratio through phosphoketolase (i.e., v_2_/v_1_ in Figure 7A) and range of effective FL are used as the selection criteria. The modeling results are shown in Figure 8.

Among various xylose tracers, 1,2-^13^C xylose yields EMU AC_12_ with best correlation between its FL and phosphoketolase flux ratio (Spearman correlation coefficient = 0.7). The widest effective range of FL (slope of regression line = 0.43) indicates a good sensitivity on the flux ratio of phosphoketolase (Figure 8A). EMU AC_1_ and AC_2_ show identical correlationship between FL and flux ratio with that of AC_12_ (Figures 8B,C) because the carbons of acetate originate from C2 and C1 of xylose which are both labeled (probably also from C4 and C5 of xylose converted from AcCoA) and behave equivalently in the atom transition. FL of G3P_123_ could have a good correlation with the phosphoketolase flux ratio, which is, however, disturbed by many randomly distributed points (Figure 8E). This is probably due to the reversibility of the reactions in glycolytic and pentose phosphate pathways. EtOH_12_, E4P_1234_ and R5P_12345_ all show poor correlation, and thus cannot be used as the indicators for phosphoketolase flux (Figures 8D,F,G). As for other tracers, 1-^13^C xylose and 2-^13^C xylose labeling result in poor correlation in AC_1_ and AC_2_, respectively (Supplementary Figures 1, 2). AC_12_ and EtOH_12_ will be totally unlabeled using 3-^13^C xylose tracer as no C3 of xylose will fractionate into these metabolites. G3P_123_ shows a good correlation with phosphoketolase flux while the range of effective FL is too small to determine phosphoketolase activity (Supplementary Figure 3). If 4-^13^C xylose and 5-^13^C xylose are used as substrate, correlation between FL and flux ratio are inverted for most of the EMUs. In addition, AC_2_ and AC_1_ are totally unlabeled per 4-^13^C xylose and 5-^13^C xylose, respectively. (Supplementary Figures 4, 5). As a control, FLs of all MDVs are constant when fed with a mixture of U-^13^C labeled and unlabeled xylose (Supplementary Figure 6), which is, therefore excluded from the isotope tracer selection. In comparison of all tracer/readout combination, 1,2-^13^C-xylose/ AC_12_ performed the best and this selection paves the way to the experimental measurement of flux in phosphoketolase pathway.

Guided by the above simulations, *C. acetobutylicum* was thereafter cultivated in 5 g L^−1^ 1,2-^13^C xylose in following experiment. Fermentation kinetics including cell growth and the production of cell products over time are as shown in Supplementary Figure 7. The flux ratio from phosphoketolase pathway was then quantified by harvesting the supernatant and determining the isotope pattern of AC with GC-MS. MDVs of AC_1_ and AC_12_ were measured, and MDVs of AC_2_ can be deduced. Accordingly, FL of AC_1_, AC_2_ and AC_12_ were calculated to be 0.462 ± 0.018, 0.469 ± 0.012 and 0.465 ± 0.015, respectively, which is consistent with our prediction that FLs of all fragments' EMUs should be identical. A distribution of phosphoketolase flux ratio was obtained from simulated sample points with corresponding FL of AC_1_, AC_2_ and AC_12_ falling into the measured ranges. The average and 95% confidence interval of the flux ratio through phosphoketolase were estimated as 22.8% and 14.3–36.6%, respectively under current conditions (Figure 7B).

## Discussion

Although equivalent to isotopomer models, EMU method is able to significantly reduce the variables needed to simulate labeling patterns of metabolites (90% reduction in our *C. acetobutylicum* case) without any loss of information, which therefore greatly facilitates ^13^C flux modeling of realistic and large-scaled metabolic network and dynamic systems (Young et al., 2008). Here we introduce a computational tool *EMUlator* to the metabolic research community. This engine utilizes adjacency matrix-based approach which is intuitively straightforward and easy to program. Metabolic network can be decomposed into EAMs of different size which can be further reduced by lumping unimolecular reactions and combining equivalent EMUs. For MDV simulation, matrix multiplication starts from EAM of the smallest size, and iteratively continues to larger size until the required EMU is simulated. Overall, the computation time for EMUlator to perform MDV simulation depends on the network complexity, or connectivity which can be represented by the number of non-zero elements in EAMs. For a realistic and moderate-sized network shown in the above examples, the time complexity is roughly *O(n*^3^*)*, where n is the number of EMUs.

The *EMUlator* allowed large-scale and efficient isotope modeling. To exemplify its capability, we applied it in quantitative understanding of the phosphoketolase pathway in the central carbon metabolism of an industrial model microbe (*C. acetobutylicum*). We simulated labeling pattern of both intracellular and secretory metabolites using different xylose tracers, and identified the best tracer/readout combination reflecting phosphoketolase activity. ^13^C-flux measurement of the phosphoketolase pathway was enabled recently (Liu et al., 2012), in which 3-phosphoglycerate was used as the indicator for phosphoketolase flux measurement in a 1-^13^C-xylose labeling experiment. This design is reasonable as the FL of 3-phosphoglycerate is monotonically reduced with the increased flux through phosphoketolase. The limitation is that 3-phosphoglycerate is not a direct product of phosphoketolase, therefore the flux estimation is largely dependent on the assumption that the labeling pattern of 3-phosphoglycerate and glyceraldehyde 3-phosphate, the product of phosphoketolase are identical. In our case, we compared all available xylose tracers and readouts with a more realistic metabolic network which also takes reversible reactions into account. Our results demonstrated that acetate (with EMUs of AC_1_, AC_2_, and AC_12_) can be a better readout due to the strong correlations with phosphoketolase flux and a wide effective range of FL. Indeed, acetate can be exclusively derived from acetyl phosphate which is directly produced from phosphoketolase. More importantly, acetate is an extracellular metabolite and its isotope pattern can be easily measured by GC-MS without derivatization. The convenience of measurement without breaking the cells provides a high-throughput and non-invasive method for prompt ^13^C-flux estimation, which, to our knowledge, was never developed previously. This estimation is based on the numerical relationship between fractional labeling of acetate and flux ratio through phosphoketolase which are positively correlated, even though it may not be strictly linear. It should be noticed that in the FL range 0.5-0.65, multiple flux ratio values are possible for a given value of FL. The jitter could be due to the reversibility of biochemical reactions and partial dependency of the EMU basis vectors (Crown and Antoniewicz, 2012), which cannot make all free fluxes absolutely solvable using only acetate labeling data. To further obtain a more precise prediction of phosphoketolase activity, advanced multiple regression method could be applied such as machine learning using FLs of other relevant EMUs as the training features.

We believe that the EMU modeling based on adjacency matrix approach opens a number of new possibilities in metabolic network analysis and ^13^C-MFA. First, as exemplified, *EMUlator* can be used to select metabolites as readouts reflecting directly *in vivo* enzyme activities, and can also be used to do tracer simulations (Metallo et al., 2009; Young, 2014) which predict the labeling results of metabolic network models ahead of “wet” experiments, leading to further refinement. More promisingly, *EMUlator* is developed toward solving the inverse problem to estimate intracellular fluxes through an optimization search that minimize the sum-of-squared residuals between computationally simulated and experimentally determined measurements. With the EMU method, metabolic flux estimation can be further extended to computation-intensive scenarios as genome scale network (Gopalakrishnan and Maranas, 2015) and transient labeling process (Hendry et al., 2019). This task cannot be accomplished by other isotope modeling methods due to a tremendous computational burden. Currently, we are engaged in the development of an updated version focusing on the *de novo* and complete solution of ^13^C-MFA, in which a global flux distribution can be estimated either from steady-state labeling or kinetic labeling experiments. It is our hope that *EMUlator* will benefit the community and fuel metabolic research as the basis for innovative development of metabolic analysis tools.

## Materials and Methods

### Implementation of EMU Algorithm in *EMUlator*

The TCA cycle example in the results illustrates the adjacency matrix approach for implementing EMU algorithm. The software and its instruction and are detailed in Supplementary Information.

### Strain, Culture Conditions, and Medium

*Clostridium acetobutylicum* ATCC 824 was used in all experiments. For growth studies and biochemical characterization, *C. acetobutylicum* cells were grown anaerobically in 37°C in CTFUD defined medium (Olson and Lynd, 2012) which contains 3 g L^−1^ Na_3_C_6_H_5_O_7_·2H_2_O, 1.3 g L^−1^ (NH_4_)_2_SO_4_, 1.5 g L^−1^ KH_2_PO_4_, 0.13 g L^−1^ CaCl_2_·2H_2_O, 0.5 g L^−1^ L-Cysteine–HCl, 11.56 g L^−1^ MOPS sodium salt, 2.6 g L^−1^ MgCl_2_·6H_2_O, 0.001 g L^−1^ FeSO_4_·7H2O, 0.5 mL L^−1^ Resazurin 0.2% (w/v), supplemented with Wolfe's Vitamin solution (ATCC). D-xylose was supplied at concentration of 5 g L^−1^ as a carbon source. The cultures were started with the optical density at 600 nm (OD_600_ = 0.05-0.08) and performed in mid-log-phase. For labeling experiments, 1,2-^13^C-labeled xylose (99% pure; Cambridge Isotope Laboratories, Tewksbury, MA) was added to media at the concentration of 5 g L^−1^. *C. acetobutylicum* strains were kept by freezing log-phase cultures at −80°C with 10% glycerol.

### Quantitative Analysis of Fermentation Products

Cell growth was monitored by measuring the absorbance at OD_600_ with a Spectronic 21D UV-Visible Spectrophotometer (Milton Roy, Houston, TX). To analyze extracellular metabolites, cell samples were harvested by centrifugation at 13,000 g for 10 min. After filtration with 0.2 μm filter, the supernatant was analyzed by Agilent 1200 high pressure liquid chromatography (HPLC) (Agilent Technologies, Santa Clara, CA) and injected into a Bio-Rad Aminex HPX-87H column with a Micro Guard Cation H Cartridge. 4 m M H_2_SO_4_ was used as mobile phase at a flow rate of 0.6 mL/min. The column temperature was set to 55°C. Metabolites were detected by refractive index detector and UV/VIS detector.

### Isotope Analysis

Labeling pattern of proteinogenic amino acids from cell mass were analyzed by Gas Chomatograph-mass spectrometry (GC-MS) as detailed in Xiong et al. (2018). The labeling pattern of acetate in the supernatant was directly analyzed by GC-MS without derivatization. Analysis of samples was performed on an Agilent 6890N GC equipped with a 5973 MS Detector (Agilent Technologies, Palo Alto, CA). Samples were injected at a volume of 1 uL in splitless mode, and the analyte of interest was separated on a Restek Stabilwax-DA column (Restek Corporation, Bellefonte, PA). A flow of 1 mL min^−1^ was held constant throughout the run with the following temperature profile: 35°C, hold for 3 min; ramped at 10 °C min^−1^ to 225°C, hold for 1 min; ramped at 15°C min^−1^ to 250°C, hold for 5 min.

### Isotope Modeling From the Metabolic Network of *C. acetobutylicum*

Simulations were repeated 1,000 times with metabolic fluxes randomly generated subjecting to mass balances determined by reactions listed in Supplementary Table 1. Fractional labeling (FL) was calculated to indicate labeling status of metabolites according to:

$$\begin{array}{l} FL\;=\;\frac{\overset{n}{\underset{i=0}{\sum }}i\cdot m_{i}}{n} \end{array}$$

where n is the number of carbon in a EMU, and m_i_ represents components of MDV (Nanchen et al., 2007).

## Author Contributions

CW developed the software and analyzed data. WX and CW designed the experiment. CC, JL, and WM conducted the experiment. WX proposed the idea and research questions, guided all stages of the research. CW and WX prepared the manuscript with editing from PM, JL, and CC.

### Conflict of Interest Statement

The authors declare that the research was conducted in the absence of any commercial or financial relationships that could be construed as a potential conflict of interest.

## References

[B1] Antoniewicz M. R. Kelleher J. K. Stephanopoulos G. (2006). Determination of confidence intervals of metabolic fluxes estimated from stable isotope measurements. Metab. Eng. 8, 324–337. 10.1016/j.ymben.2006.01.004 16631402

[B2] Antoniewicz M. R. Kelleher J. K. Stephanopoulos G. (2007a). Elementary metabolite units (EMU): a novel framework for modeling isotopic distributions. Metab. Eng. 9, 68–86. 10.1016/j.ymben.2006.09.001 17088092 PMC1994654

[B3] Antoniewicz M. R. Kraynie D. F. Laffend L. A. González-Lergier J. Kelleher J. K. Stephanopoulos G. (2007b). Metabolic flux analysis in a nonstationary system: fed-batch fermentation of a high yielding strain of *E. coli* producing 1,3-propanediol. Metab. Eng. 9, 277–292. 10.1016/j.ymben.2007.01.003 17400499 PMC2048574

[B4] Au J. Choi J. Jones S. W. Venkataramanan K. P. Antoniewicz M. R. (2014). Parallel labeling experiments validate *Clostridium acetobutylicum* metabolic network model for (13)C metabolic flux analysis. Metab. Eng. 26, 23–33. 10.1016/j.ymben.2014.08.002 25183671

[B5] Bao G. Wang R. Zhu Y. Dong H. Mao S. Zhang Y.. (2011). Complete genome sequence of Clostridium acetobutylicum DSM 1731, a solvent-producing strain with multireplicon genome architecture. J. Bacteriol. 193, 5007–5008. 10.1128/JB.05596-11 21742891 PMC3165653

[B6] Bogorad I. W. Lin T. S. Liao J. C. (2013). Synthetic non-oxidative glycolysis enables complete carbon conservation. Nature 502, 693–697. 10.1038/nature12575 24077099

[B7] Crown S. B. Antoniewicz M. R. (2012). Selection of tracers for 13C-metabolic flux analysis using elementary metabolite units (EMU) basis vector methodology. Metab. Eng. 14, 150–161. 10.1016/j.ymben.2011.12.005 22209989 PMC6474252

[B8] Gopalakrishnan S. Maranas C. D. (2015). 13C metabolic flux analysis at a genome-scale. Metab. Eng. 32, 12–22. 10.1016/j.ymben.2015.08.006 26358840

[B9] Grimmler C. Held C. Liebl W. Ehrenreich A. (2010). Transcriptional analysis of catabolite repression in Clostridium acetobutylicum growing on mixtures of D-glucose and D-xylose. J. Biotechnol. 150, 315–323. 10.1016/j.jbiotec.2010.09.938 20883732

[B10] Gu Y. Jiang Y. Wu H. Liu X. Li Z. Li J.. (2011). Economical challenges to microbial producers of butanol: feedstock, butanol ratio and titer. Biotechnol. J. 6, 1348–1357. 10.1002/biot.201100046 22076745

[B11] Hendry J. I. Gopalakrishnan S. Ungerer J. Pakrasi H. B. Tang Y. J. Maranas C. D. (2019). Genome-scale fluxome of *Synechococcus elongatus* UTEX 2973 Using Transient ^13^C-labeling data. Plant Physiol. 179, 761–769. 10.1104/pp.18.01357 30552197 PMC6367904

[B12] Kajihata S. Furusawa C. Matsuda F. Shimizu H. (2014). OpenMebius: an open source software for isotopically nonstationary 13C-based metabolic flux analysis. Biomed. Res. Int. 2014:627014. 10.1155/2014/627014 25006579 PMC4071984

[B13] Liu L. Zhang L. Tang W. Gu Y. Hua Q. Yang S.. (2012). Phosphoketolase pathway for xylose catabolism in Clostridium acetobutylicum revealed by 13C metabolic flux analysis. J. Bacteriol. 194, 5413–5422. 10.1128/JB.00713-12 22865845 PMC3457242

[B14] Metallo C. M. Walther J. L. Stephanopoulos G. (2009). Evaluation of 13C isotopic tracers for metabolic flux analysis in mammalian cells. J. Biotechnol. 144, 167–174. 10.1016/j.jbiotec.2009.07.010 19622376 PMC3026314

[B15] Mitchell W. J. (1998). Physiology of carbohydrate to solvent conversion by clostridia. Adv. Microb. Physiol. 39, 31–130. 10.1016/S0065-2911(08)60015-6 9328646

[B16] Nanchen A. Fuhrer T. Sauer U. (2007). Determination of metabolic flux ratios from 13C-experiments and gas chromatography-mass spectrometry data: protocol and principles. Methods Mol. Biol. 358, 177–197. 10.1007/978-1-59745-244-1_11 17035687

[B17] Nölling J. Breton G. Omelchenko M. V. Makarova K. S. Zeng Q. Gibson R.. (2001). Genome sequence and comparative analysis of the solvent-producing bacterium Clostridium acetobutylicum. J. Bacteriol. 183, 4823–4838. 10.1128/JB.183.16.4823-4838.2001 11466286 PMC99537

[B18] Olson D. G. Lynd L. R. (2012). Transformation of *Clostridium thermocellum* by electroporation. Methods Enzymol. 510, 317–330. 10.1016/B978-0-12-415931-0.00017-3 22608734

[B19] Quek L. E. Wittmann C. Nielsen L. K. Krömer J. O. (2009). OpenFLUX: efficient modelling software for 13C-based metabolic flux analysis. Microb. Cell Fact. 8, 25. 10.1186/1475-2859-8-25 19409084 PMC2689189

[B20] Sauer U. (2006). Metabolic networks in motion: 13C-based flux analysis. Mol. Syst. Biol. 2, 62. 10.1038/msb4100109 17102807 PMC1682028

[B21] Schmidt K. Carlsen M. Nielsen J. Villadsen J. (1997). Modeling isotopomer distributions in biochemical networks using isotopomer mapping matrices. Biotechnol. Bioeng. 55, 831–840. 18636594 10.1002/(SICI)1097-0290(19970920)55:6<831::AID-BIT2>3.0.CO;2-H

[B22] Servinsky M. D. Kiel J. T. Dupuy N. F. Sund C. J. (2010). Transcriptional analysis of differential carbohydrate utilization by *Clostridium acetobutylicum*. Microbiology 156, 3478–3491. 10.1099/mic.0.037085-0 20656779

[B23] Shupletsov M. S. Golubeva L. I. Rubina S. S. Podvyaznikov D. A. Iwatani S. Mashko S. V. (2014). OpenFLUX2: (13)C-MFA modeling software package adjusted for the comprehensive analysis of single and parallel labeling experiments. Microb. Cell. Fact. 13, 152. 10.1186/PREACCEPT-1256381938128538 25408234 PMC4263107

[B24] Sokol S. Millard P. Portais J. C. (2012). influx_s: increasing numerical stability and precision for metabolic flux analysis in isotope labelling experiments. Bioinformatics 28, 687–693. 10.1093/bioinformatics/btr716 22210866

[B25] Stephanopoulos G. (1999). Metabolic fluxes and metabolic engineering. Metab Eng. 1, 1–11. 10935750 10.1006/mben.1998.0101

[B26] Van Winden W. A. Heijnen J. J. Verheijen P. J. (2002). Cumulative bondomers: a new concept in flux analysis from 2D [13C,1H] COSY NMR data. Biotechnol. Bioeng. 80, 731–745. 10.1002/bit.10429 12402319

[B27] Weitzel M. Nöh K. Dalman T. Niedenführ S. Stute B. Wiechert W. (2013). 13CFLUX2–high-performance software suite for (13)C-metabolic flux analysis. Bioinformatics 29, 143–145. 10.1093/bioinformatics/bts646 23110970 PMC3530911

[B28] Wiechert W. Möllney M. Isermann N. Wurzel M. De Graaf A. A. (1999). Bidirectional reaction steps in metabolic networks: III. Explicit solution and analysis of isotopomer labeling systems. Biotechnol. Bioeng. 66, 69–85. 10567066

[B29] Xiong W. Lo J. Chou K. J. Wu C. Magnusson L. Dong T.. (2018). Isotope-assisted metabolite analysis sheds light on central carbon metabolism of a model cellulolytic bacterium clostridium thermocellum. Front. Microbiol. 9:1947. 10.3389/fmicb.2018.01947 30190711 PMC6115520

[B30] Young J. D. (2014). INCA: a computational platform for isotopically non-stationary metabolic flux analysis. Bioinformatics 30, 1333–1335. 10.1093/bioinformatics/btu015 24413674 PMC3998137

[B31] Young J. D. Walther J. L. Antoniewicz M. R. Yoo H. Stephanopoulos G. (2008). An elementary metabolite unit (EMU) based method of isotopically nonstationary flux analysis. Biotechnol. Bioeng. 99, 686–699. 10.1002/bit.21632 17787013

