# Supplementary material for: EMUlator: An Elementary Metabolite Unit (EMU) Based Isotope Simulator Enabled by Adjacency Matrix
Source: Front Microbiol. 2019 Apr 30;10:922. doi: 10.3389/fmicb.2019.00922 (PMC6503117; doi:10.3389/fmicb.2019.00922)
Supplement: Supplementary file 1 [file Data_Sheet_1.docx]

**Supplementary Table 1**. Biochemical reactions as well as atom transitions involved in xylose metabolism of *C. acetobutylicum*.

| **Reaction ID** | **Reaction with atom transition** |
| --- | --- |
| v1 | Xyl(abcde) => X5P(abcde) |
| v2 | X5P(abcde) => AcP(ba)+GAP(cde) |
| v3 | X5P(abcde) <=> Ru5P(abcde) |
| v4 | Ru5P(abcde) <=> R5P(abcde) |
| v5 | X5P(abcde) <=> TK(ab) + GAP(cde) |
| v6 | F6P(abcdef) <=> TK(ab) + E4P(cdef) |
| v7 | S7P(abcdefg) <=> TK(ab) + R5P(cdefg) |
| v8 | F6P(abcdef) <=> TA(abc) + GAP(def) |
| v9 | S7P(abcdefg) <=> TA(abc) + E4P(defg) |
| v10 | F6P(abcdef) => FBP(abcdef) |
| v11 | FBP(abcdef) <=> DHAP(cba)+GAP(def) |
| v12 | DHAP(abc) <=> GAP(abc) |
| v13 | GAP(abc) <=> G3P(abc) |
| v14 | G3P(abc) <=> PEP(abc) |
| v15 | PEP(abc) => Pyr(abc) |
| v16 | Pyr(abc) => AcCoA(bc) + CO_2_(a) |
| v17 | AcCoA(ab) <=> AcP(ab) |
| v18 | AcP(ab) => AC(ab) |
| v19 | AcCoA(ab) => EtOH(ab) |

(A) (B)


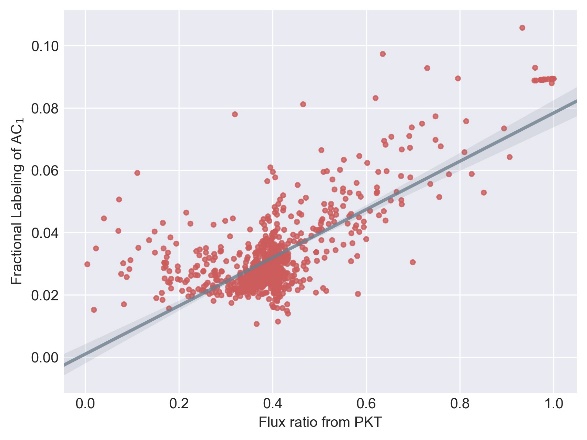

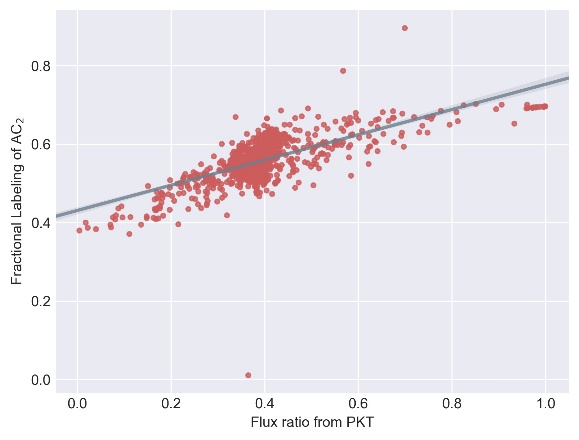


(C) (D)


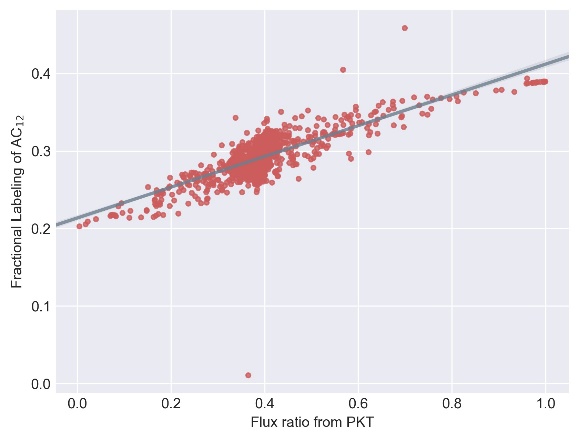

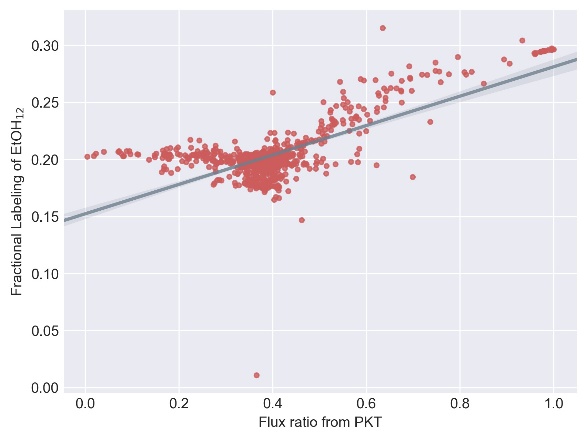


(E) (F)


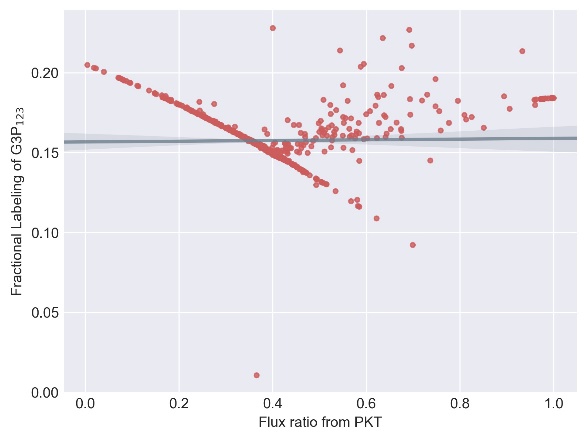

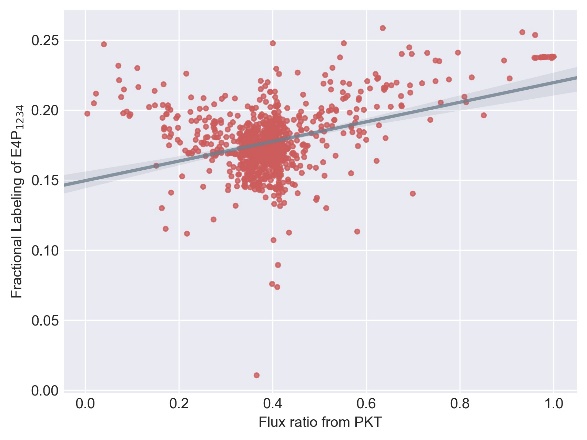


(G)


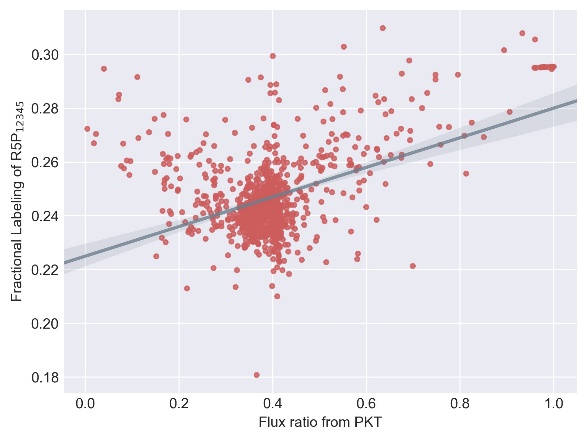


**Supplementary Figure 1**.

Simulated FL of metabolites at different flux ratio from PKT with 100% 1-^13^C xylose as substrate. Random fluxes are generated 1000 times subjecting to xylose metabolism network. MDVs of (A) AC_1_, (B) AC_2_, (C) AC_12_, (D) EtOH_12_, (E) G3P_123_, (F) E4P_1234_ and (G) R5P_12345_ are simulated using adjacency matrix based EMU decomposition method proposed in this work. Metabolite FLs and flux ratio from PKT are subsequently calculated and plotted correspondingly. Regression line and 95% confidence intervals are also plotted.

(A) (B)


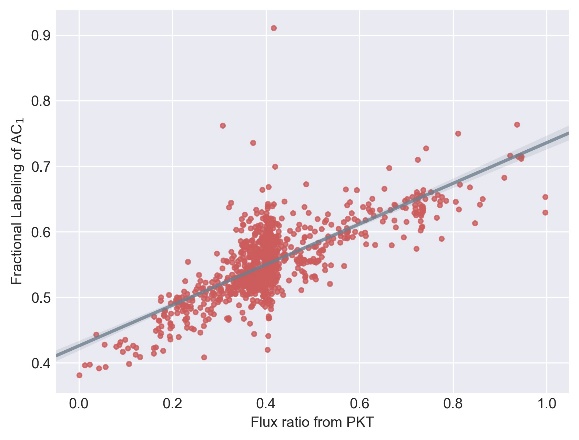

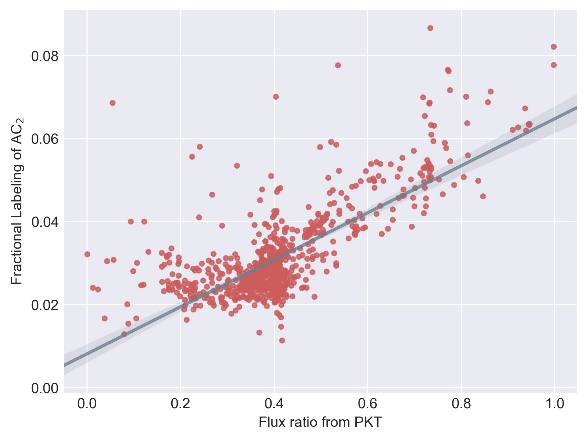


(C) (D)


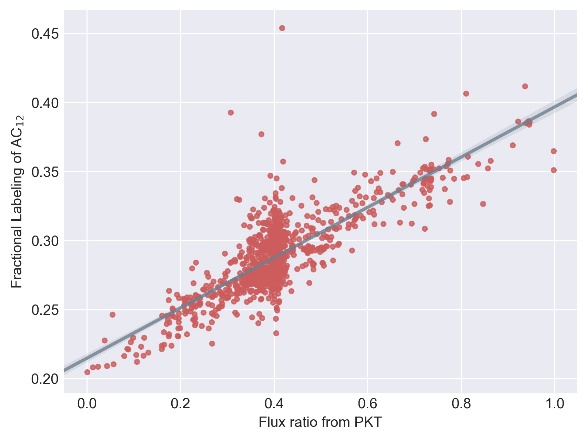

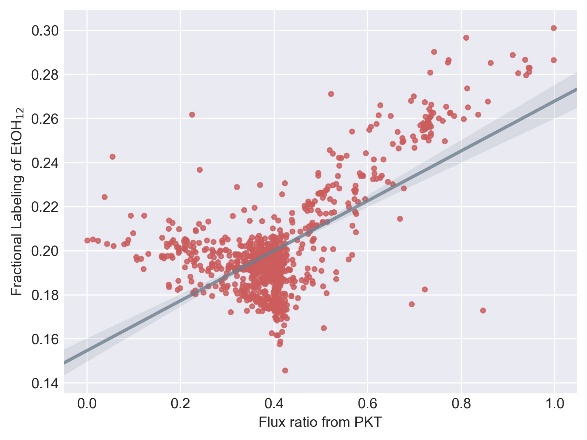


(E) (F)


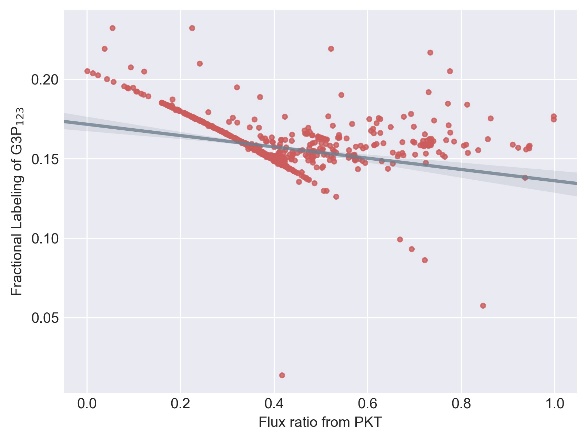

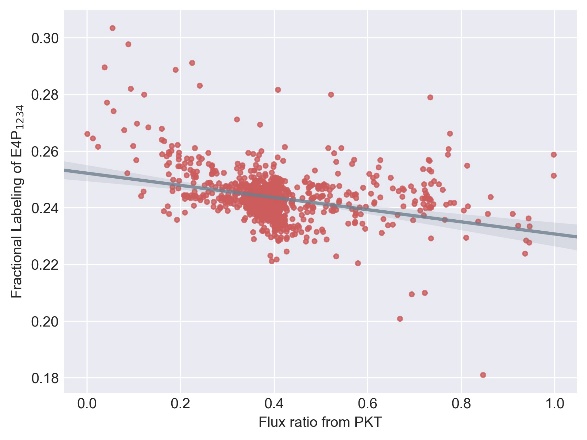


(G)


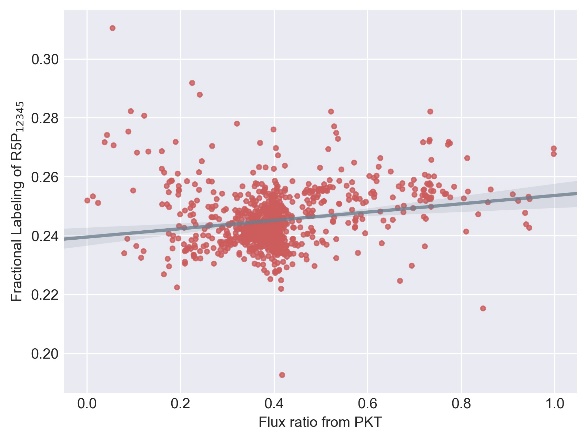


**Supplementary Figure 2**.

Simulated FL of metabolites at different flux ratio from PKT with 100% 2-^13^C xylose as substrate. Random fluxes are generated 1000 times subjecting to xylose metabolism network. MDVs of (A) AC_1_, (B) AC_2_, (C) AC_12_, (D) EtOH_12_, (E) G3P_123_, (F) E4P_1234_ and (G) R5P_12345_ are simulated using adjacency matrix based EMU decomposition method proposed in this work. Metabolite FLs and flux ratio from PKT are subsequently calculated and plotted correspondingly. Regression line and 95% confidence intervals are also plotted.

(A) (B)


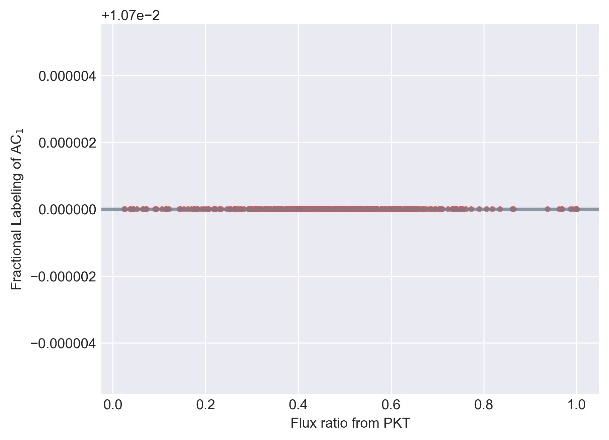

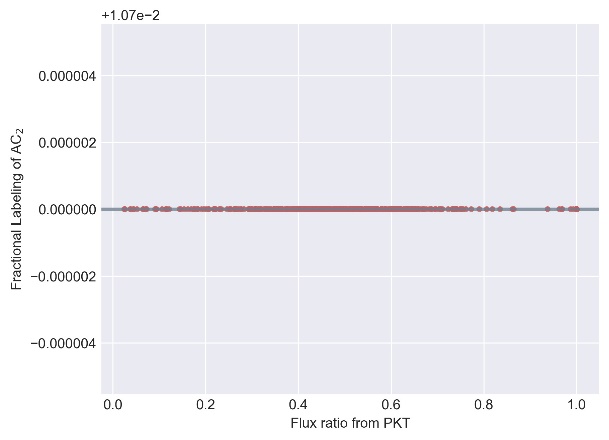


(C) (D)


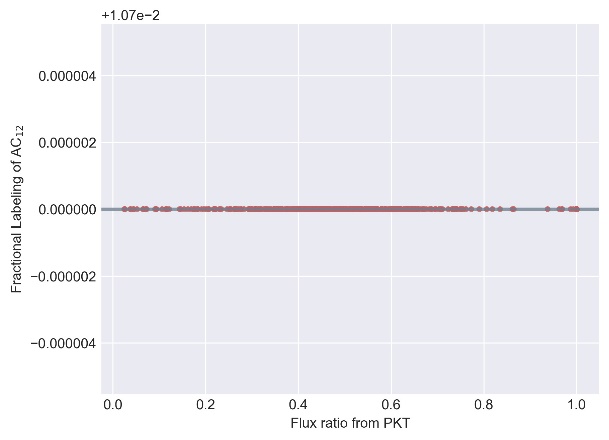

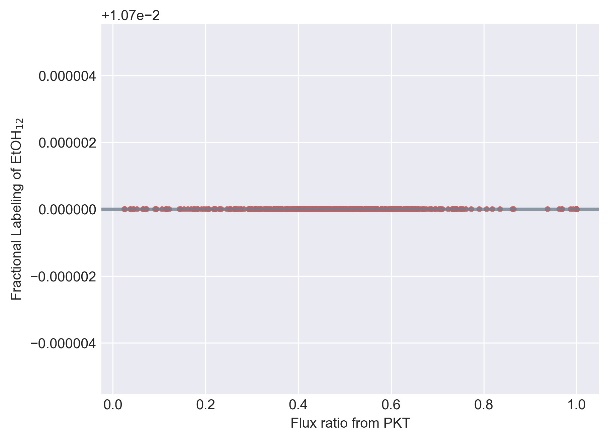


(E) (F)


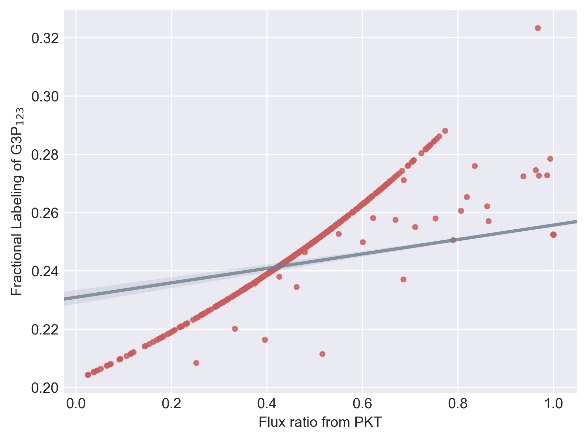

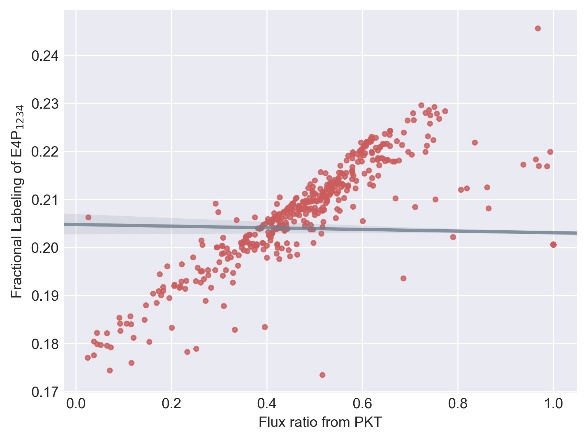


(G)


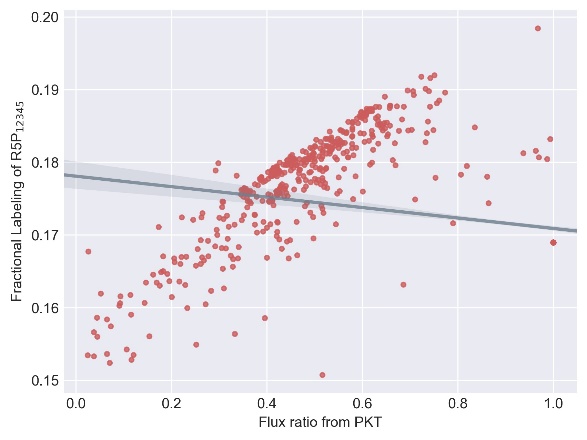


**Supplementary Figure 3**.

Simulated FL of metabolites at different flux ratio from PKT with 100% 3-^13^C xylose as substrate. Random fluxes are generated 1000 times subjecting to xylose metabolism network. MDVs of (A) AC_1_, (B) AC_2_, (C) AC_12_, (D) EtOH_12_, (E) G3P_123_, (F) E4P_1234_ and (G) R5P_12345_ are simulated using adjacency matrix based EMU decomposition method proposed in this work. Metabolite FLs and flux ratio from PKT are subsequently calculated and plotted correspondingly. Regression line and 95% confidence intervals are also plotted.

(A) (B)


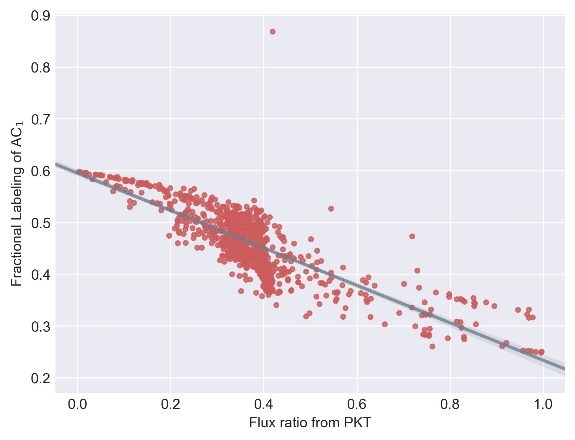

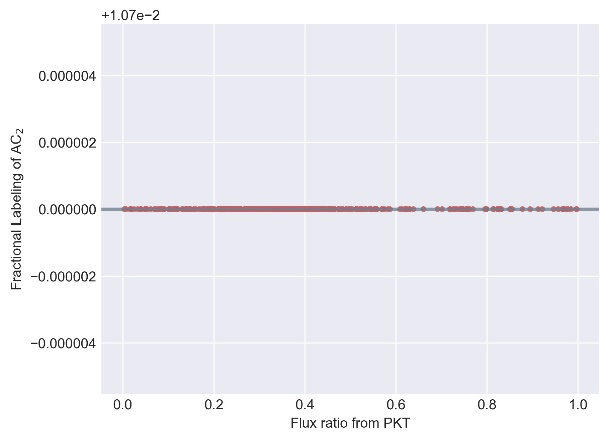


(C) (D)


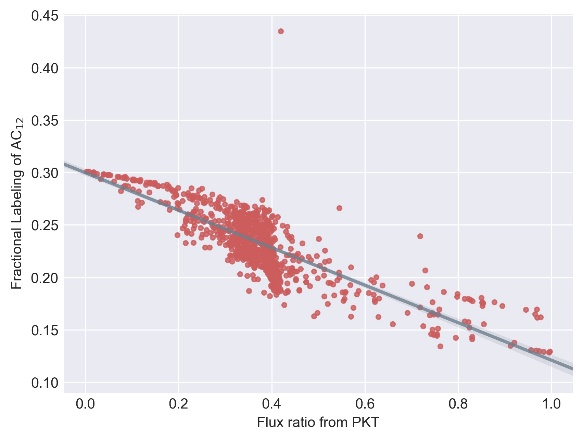

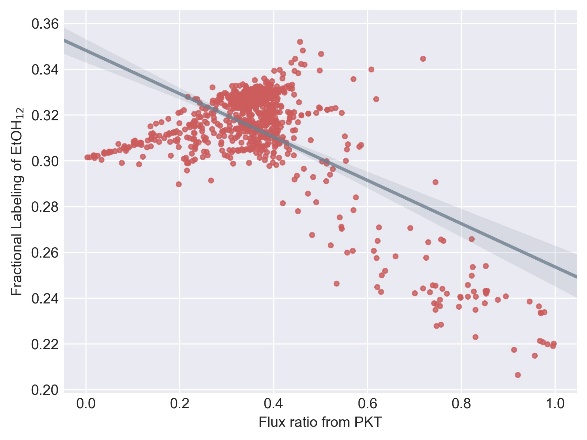


(E) (F)


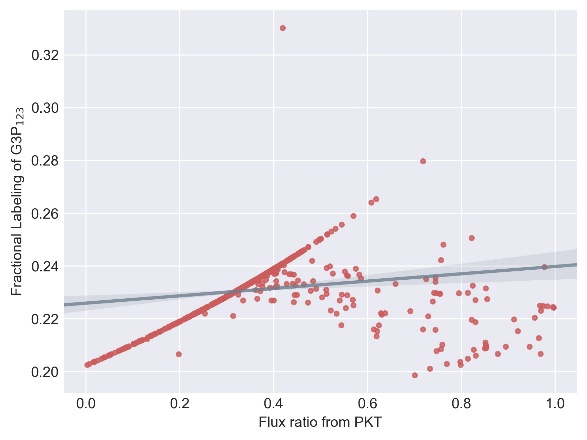

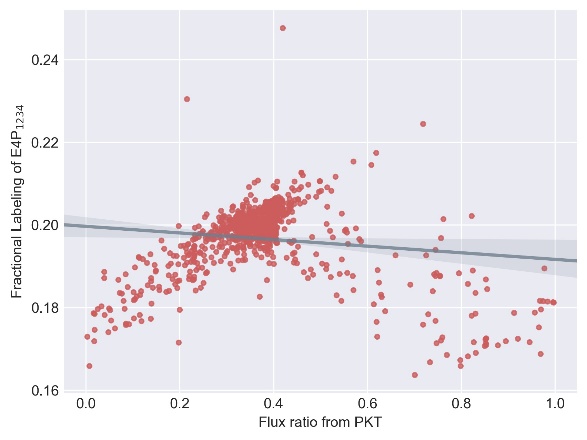


(G)


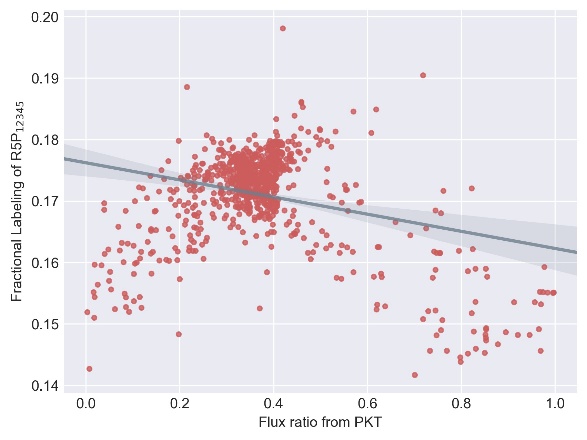


**Supplementary Figure 4**.

Simulated FL of metabolites at different flux ratio from PKT with 100% 4-^13^C xylose as substrate. Random fluxes are generated 1000 times subjecting to xylose metabolism network. MDVs of (A) AC_1_, (B) AC_2_, (C) AC_12_, (D) EtOH_12_, (E) G3P_123_, (F) E4P_1234_ and (G) R5P_12345_ are simulated using adjacency matrix based EMU decomposition method proposed in this work. Metabolite FLs and flux ratio from PKT are subsequently calculated and plotted correspondingly. Regression line and 95% confidence intervals are also plotted.

(A) (B)


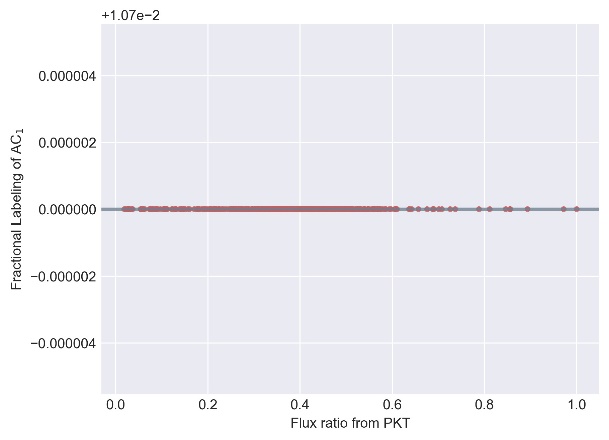

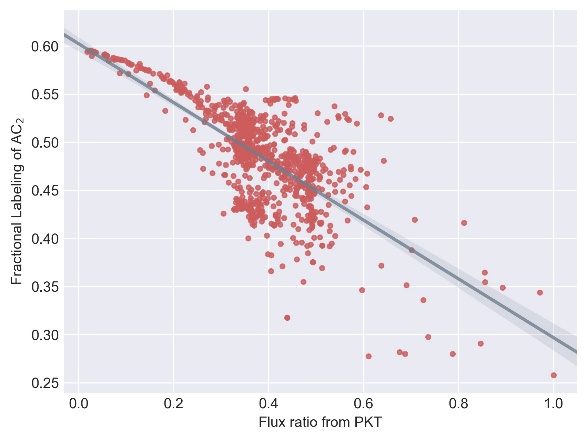


(C) (D)


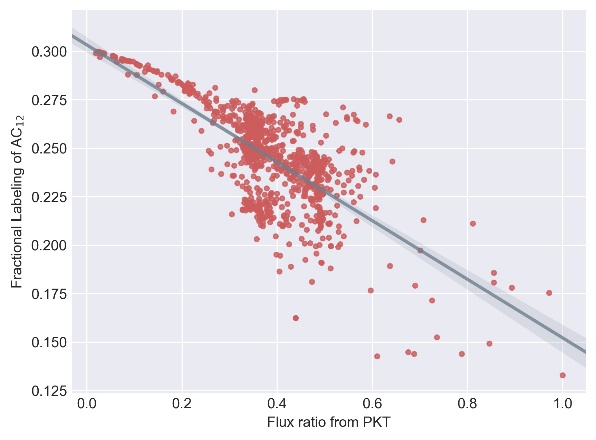

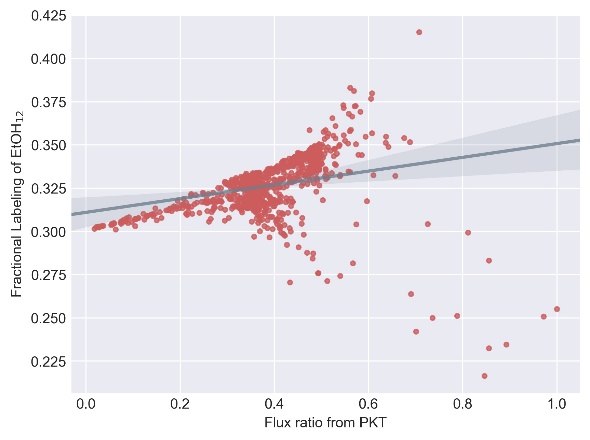


(E) (F)


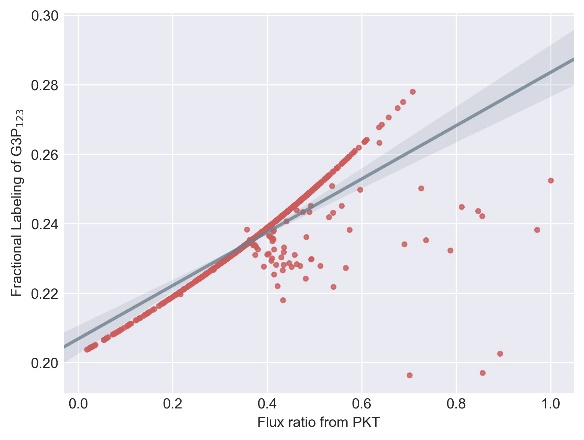

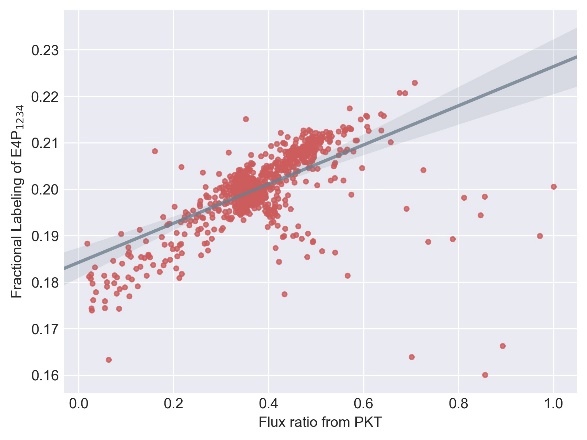


(G)


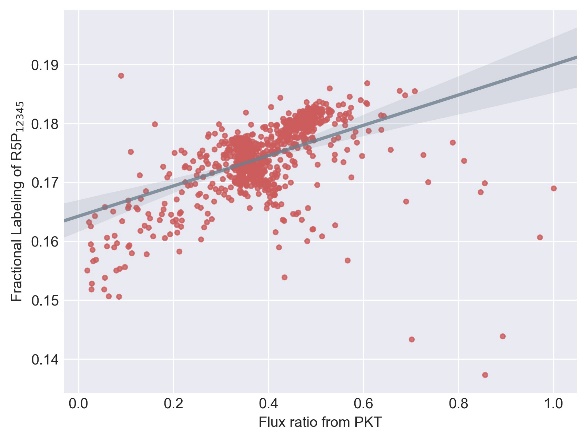


**Supplementary Figure 5**.

Simulated FL of metabolites at different flux ratio from PKT with 100% 5-^13^C xylose as substrate. Random fluxes are generated 1000 times subjecting to xylose metabolism network. MDVs of (A) AC_1_, (B) AC_2_, (C) AC_12_, (D) EtOH_12_, (E) G3P_123_, (F) E4P_1234_ and (G) R5P_12345_ are simulated using adjacency matrix based EMU decomposition method proposed in this work. Metabolite FLs and flux ratio from PKT are subsequently calculated and plotted correspondingly. Regression line and 95% confidence intervals are also plotted.

(A) (B)


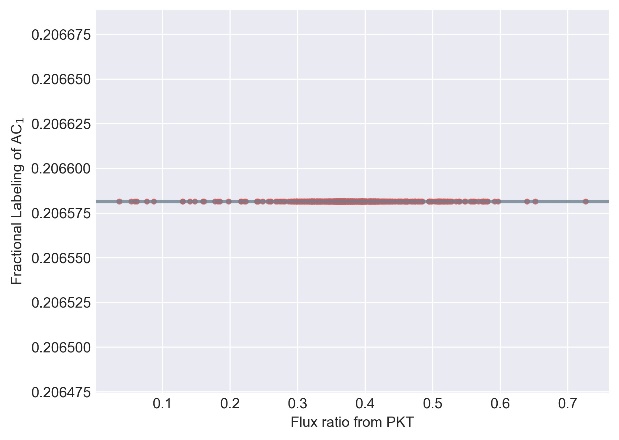

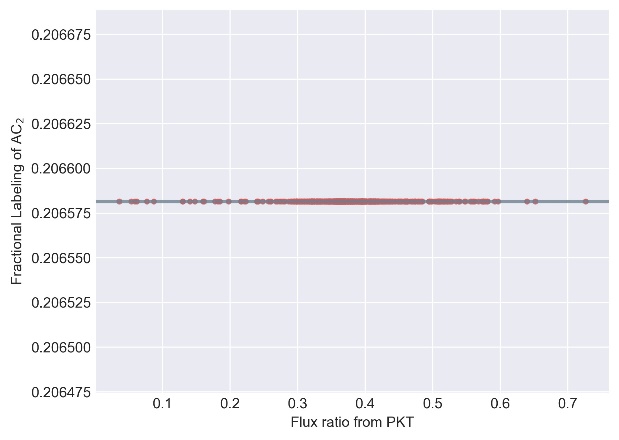


(C) (D)


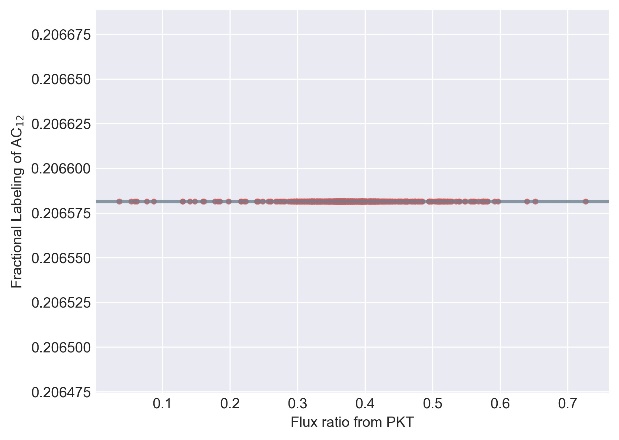

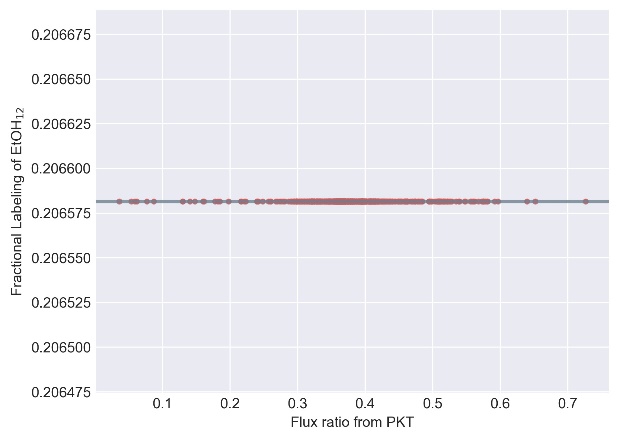


(E) (F)


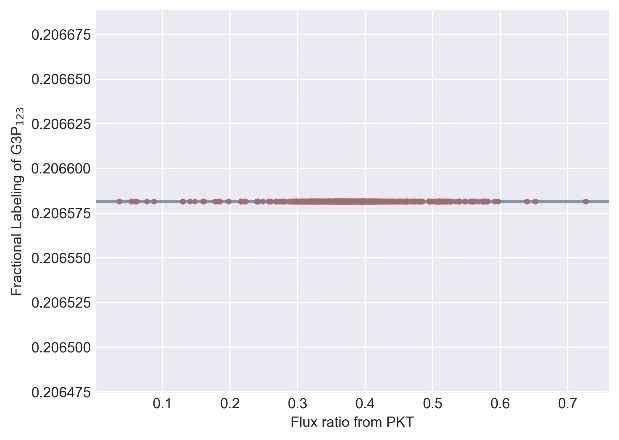

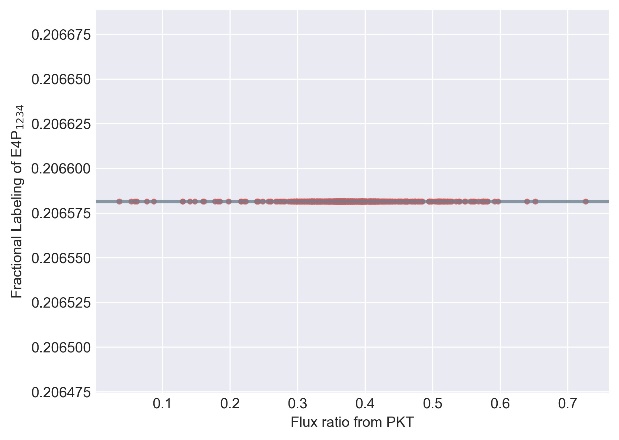


(G)


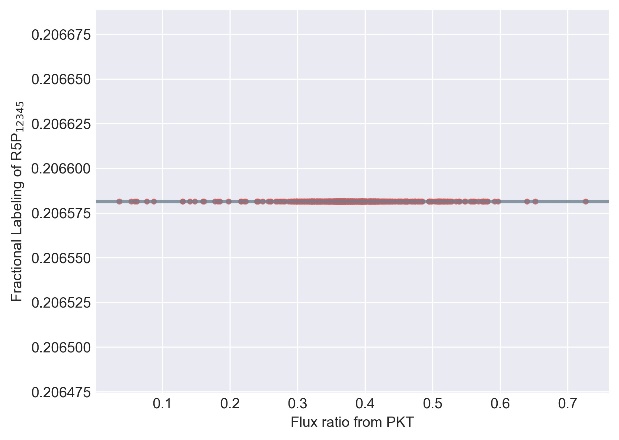


**Supplementary Figure 6**.

Simulated FL of metabolites at different flux ratio from PKT with mixture of 20% U-^13^C xylose and 80% natural xylose as substrate. Random fluxes are generated 1000 times subjecting to xylose metabolism network. MDVs of (A) AC_1_, (B) AC_2_, (C) AC_12_, (D) EtOH_12_, (E) G3P_123_, (F) E4P_1234_ and (G) R5P_12345_ are simulated using adjacency matrix based EMU decomposition method proposed in this work. Metabolite FLs and flux ratio from PKT are subsequently calculated and plotted correspondingly. Regression line and 95% confidence intervals are also plotted.


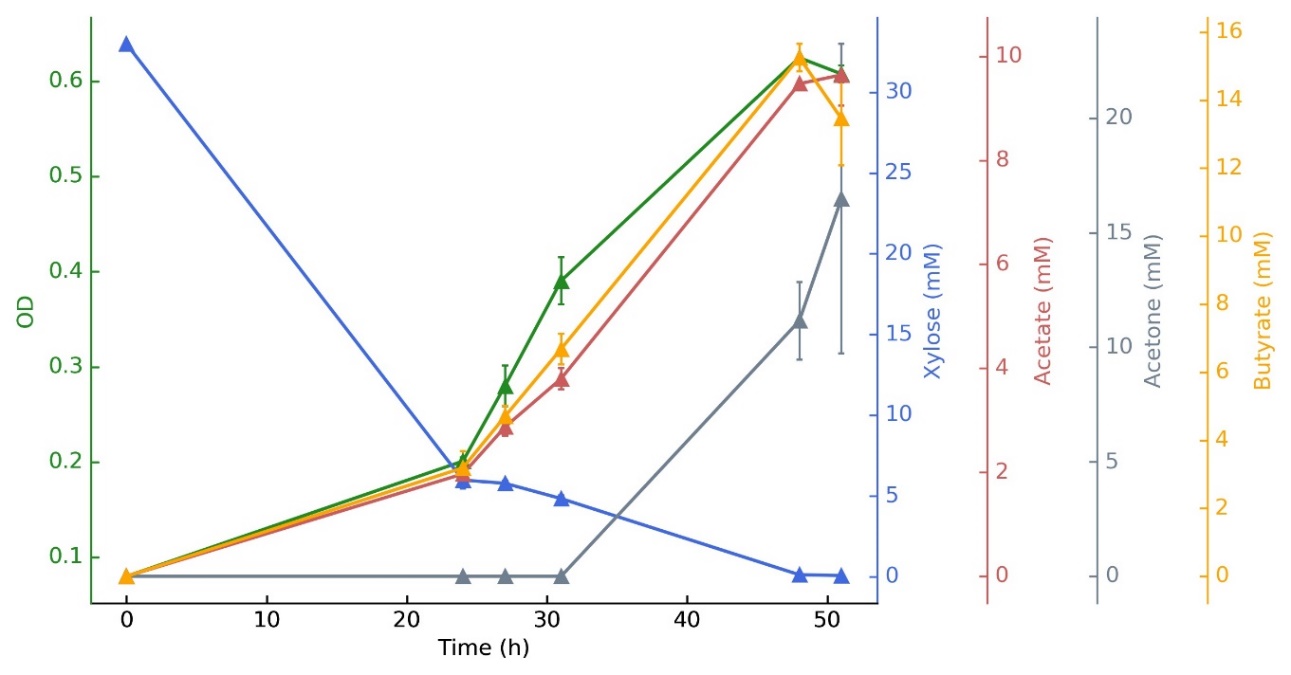


**Supplementary Figure 7**.

Cell Growth, substrate consumption and products formation of *C. acetobutylicum* at an initial xylose concentration of 5 g L^-1^. OD (green), xylose (blue), acetate (red), acetone (grey) and butyrate (yellow) are determined during cultivation. Data points represent the mean of three replicates.
