# Supplementary material for: EMUlator: An Elementary Metabolite Unit (EMU) Based Isotope Simulator Enabled by Adjacency Matrix
Source: Front Microbiol. 2019 Apr 30;10:922. doi: 10.3389/fmicb.2019.00922 (PMC6503117; doi:10.3389/fmicb.2019.00922)
Supplement: Supplementary file 2 [file Data_Sheet_2.docx]

**EMUlator user manual**

EMUlator is a python-based software package for elementary metabolite unit (EMU) decomposition of given metabolic networks. The decomposition is implemented using an adjacency matrix method we proposed in this work which is intuitively straightforward and easy to program. The software also simulates mass distribution vectors (MDVs) of intermediates under arbitrary metabolic flux distributions subject to mass balance, and further simulates relationship between split ratios of diverging pathways and fractional labeling (FL) of corresponding metabolites. The software facilities design of ^13^C-tracer experiment and selection of easily-measured readouts to illustrate activity of intracellular pathways of interest, and also can be used to solve the inverse problem of estimating intracellular fluxes through an optimization search that minimize the sum-of-squared residuals between simulated and experimentally determined measurements. The software runs in Windows command line (DOS), and can be accelerated by simulating metabolites in parallel for mutli-core machines. Basic information and usage of this software are provided as below:

**Platform**

The software was tested on Windows10.

**Development language**

The software was developed using Python3.6.

**Module dependencies**

The following python packages are required which can be installed independently with pip or integrally deployed using Anaconda (recommended):

numpy1.14.3, pandas0.22.0, scipy1.1.0, matplotlib2.1.2, seaborn0.8.1, sklearn0.19.1 and pygam0.5.4

**Flowchart**


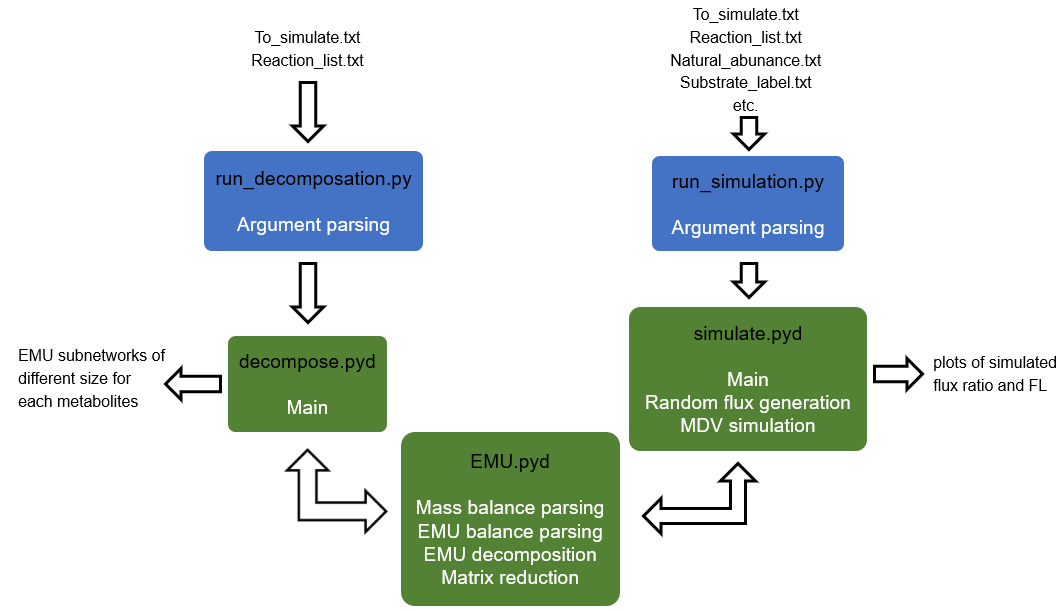


Figure 1. Flowchart of EMUlator pipeline

**Usage**

**1 EMU decomposition**

**Run script:**

run_decomposation.py

**Inputs:**

-o, --output_dir:

output directory.

-c, --sim_file:

file of EMU(s) to simulate. One EMU per line.

-r, --reaction_file:

file of reactions. The file consists of four columns separated by TAB. First column is reaction_ID. reaction IDs are composed of any alphabetic, numeric or underscore characters, while start only with letters. Second column is reactant_IDs which represents all reactants in current reaction. reactant IDs are composed of any alphabetic, numeric or underscore characters, while start/end only with letters. Multiple reactants are joined with ‘+’. Lowercase letters in parentheses represents atom transitions. Reactants without parentheses are not involved in EMU balance. Third column is product_IDs with the same format with reactant_IDs. Forth column is reaction reversibility. 0 represents irreversible, and 1 represents reversible reaction.

-h, --help:

display help information.

**Outputs:**

------ output directory

|

|------ EMU to decompose

| |

| |------ EMU adjacency matrix (EAM) of size 1

| |

| |------ EAM of other sizes…

|

|------ other EMUs to decompose …

**Example:**

python C:\Users\cwu\Desktop\EMUlator\run_decomposation.py -o C:\Users\cwu\Desktop\EMUlator\test -c C:\Users\cwu\Desktop\EMUlator\example\TCA_cycle\To_simulate.txt -r C:\Users\cwu\Desktop\EMUlator\example\TCA_cycle\Reaction_list.txt

**2 Simulation of FL and flux ratio**

**Run script:**

run_simulation.py

**Inputs:**

-o, --output_dir:

same as above.

-c, --sim_file:

same as above.

-r, --reaction_file:

same as above.

-a, --abun_file:

file of natural abundance of atoms for correction. Fields are separated by TAB.

-s, --subs_file:

file indicating the labeling pattern of substrates. There are four columns, as substrate ID, fractions, purity and labeling pattern. Labeling pattern is expressed in 01 combination delimited by ‘,’. 0 represents ^12^C atom, while 1 represents ^13^C atom. For example, 1,2-^13^C-glucose can be expressed as 1,1,0,0,0,0. Fields are separated by TAB.

-e, --exclude_file:

file of metabolites that are excluded from mass balance. One EMU per line. ‘none’ if no such metabolite.

-n, --n_sim:

number of random flux distributions.

-m, --ratio_name:

name of the flux ratio to be simulated.

-t, --reactions:

fluxes that define the ratio, delimited by ‘,’. For example, ‘v1,v2’ represents flux ratio of v1/v2.

-w, --fitting_method:

method to fit the simulated data after ploting, which should be ‘linear’ (representing linear regression) or ‘gam’ (representing generalized additive model)

-p, --n_process:

number of processes that run in parallel. It should be no greater than the total number of CPU cores.

**Outputs:**

------ output directory

|

|------ plot of flux ratio and FL of EMU

|

|------ plot of flux ratio and FL of other EMUs …

|

|------ simulated.tsv
